# Pea Broth Enhances the Biocontrol Efficacy of *Lysobacter capsici* AZ78 by Triggering Cell Motility Associated with Biogenesis of Type IV Pilus

**DOI:** 10.3389/fmicb.2016.01136

**Published:** 2016-07-26

**Authors:** Selena Tomada, Gerardo Puopolo, Michele Perazzolli, Rita Musetti, Nazia Loi, Ilaria Pertot

**Affiliations:** ^1^Department of Sustainable Agro-Ecosystems and Bioresources, Research and Innovation Centre, Fondazione Edmund MachSan Michele all’Adige, Italy; ^2^Agricultural Science and Biotechnology Program, Department of Agricultural, Food, Environmental, and Animal Sciences, University of UdineUdine, Italy; ^3^Department of Agricultural, Food, Environmental, and Animal Sciences, University of UdineUdine, Italy

**Keywords:** *Lysobacter*, plant components, type IV pilus, flagellum, biological control

## Abstract

Bacterial cells can display different types of motility, due to the presence of external appendages such as flagella and type IV pili. To date, little information on the mechanisms involved in the motility of the *Lysobacter* species has been available. Recently, *L. capsici* AZ78, a biocontrol agent of phytopathogenic oomycetes, showed the ability to move on jellified pea broth. Pea broth medium improved also the biocontrol activity of *L. capsici* AZ78 against *Plasmopara viticola* under greenhouse conditions. Noteworthy, the quantity of pea residues remaining on grapevine leaves fostered cell motility in *L. capsici* AZ78. Based on these results, this unusual motility related to the composition of the growth medium was investigated in bacterial strains belonging to several *Lysobacter* species. The six *L. capsici* strains tested developed dendrite-like colonies when grown on jellified pea broth, while the development of dendrite-like colonies was not recorded in the media commonly used in motility assays. To determine the presence of genes responsible for biogenesis of the flagellum and type IV pili, the genome of *L. capsici* AZ78 was mined. Genes encoding structural components and regulatory factors of type IV pili were upregulated in *L. capsici* AZ78 cells grown on the above-mentioned medium, as compared with the other tested media. These results provide new insight into the motility mechanism of *L. capsici* members and the role of type IV pili and pea compounds on the epiphytic fitness and biocontrol features of *L. capsici* AZ78.

## Introduction

Motility is a key trait that allows bacteria to reach nutrients, colonize natural niches and display pathogenic and antagonistic aptitudes (Dörr et al., 1998; Harshey, 2003). Henrichsen (1972) first classified bacterial motility into six distinct types, namely darting, gliding, spreading, swarming, swimming, and twitching. Whereas darting and spreading indicated different forms of passive dispersal of bacterial cells, the other motility types indicated active propagation of bacterial cells on inert surfaces (Henrichsen, 1972). Swimming motility indicates bacterial cell dispersal that is dependent on the presence of rotating flagella and allows bacteria to move in environments characterized by a high water content (Harshey, 2003; Kearns, 2010). Swarming motility is a dispersal mechanism for bacterial cells on semi-solid surfaces; it is made possible by functional rotating flagella, and in some cases by secreted amphiphilic compounds (surfactants) that reduce the tension between bacterial cells and inert surfaces (Kearns, 2010). Surfactins and rhamnolipids are examples of surfactants involved in the swarming motility of strains belonging to *Bacillus subtilis* and *Pseudomonas aeruginosa*, respectively (Caiazza et al., 2005; Julkowska et al., 2005). Additional external cell appendages can also be involved in swarming motility, such as type IV pili (T4P). Köhler et al. (2000) proved that T4P play a key role in the swarming motility of *P. aeruginosa* PAO1, suggesting that bacterial swarming motility is not exclusively flagella-dependent. Furthermore, the involvement of T4P in swarming motility was also confirmed in the phytopathogenic bacterial strain *P. syringae* pv. *tabaci* 6605 (Taguchi and Ichinose, 2011). However, the extension and retraction of T4P are considered key processes, generally associated with the twitching motility observed in several bacterial species, such as *P. aeruginosa*, *Ralstonia solanacearum*, and *Vibrio cholerae* (Mattick, 2002). Henrichsen (1972) also introduced another type of motility, called gliding, which does not need the presence of flagella and T4P, and originates from movement along the long cell axis characterizing bacterial strains belonging to the *Cytophaga* and *Flavobacterium* genera (McBride, 2001; Kearns, 2010). However, the involvement of T4P has been demonstrated in so-called ‘social gliding’ in some bacterial species, as in the case of *Mixococcus xanthus* (Wu et al., 1997, 1998; McBride, 2001). Movement on surfaces through gliding motility was also attributed to bacteria belonging to the genus *Lysobacter*, and most of the bacterial species in this genus lack flagella (Christensen and Cook, 1978; Hayward et al., 2010). However, the presence of a single polar flagellum was reported for some recently proposed new species, such as *L. spongiicola*, *L. arseniciresistens*, and *L. mobilis* (Romanenko et al., 2008; Luo et al., 2012; Yang et al., 2015).

Sequencing and annotation of the genomes of *Lysobacter* strains shed light on the presence of the genes involved in the biogenesis of flagella and T4P in this bacterial genus (de Bruijn et al., 2015; Kwak et al., 2015; Liu et al., 2015). The presence of genes related to the flagellar machinery was reported in the genome of strains belonging to *L. arseniciresistens*, *L. capsici*, *L. enzymogenes*, and *L. gummosus* (Wang et al., 2014; de Bruijn et al., 2015; Liu et al., 2015). However, the biosynthesis pathway of the flagellum was not functional in some strains, such as *L. capsici* 55, *L. enzymogenes* C3, and *L. gummosus* 3.2.11 (de Bruijn et al., 2015). Moreover, the genomes of *L. antibioticus* ATCC 29479, *L. antibioticus* 76, *L. capsici* 55, *L. dokdonensis* DS-58^T^, *L. enzymogenes* C3, *L. enzymogenes* OH11, and *L. gummosus* 3.2.11 encompass several genes involved in T4P biogenesis (Patel et al., 2011; Wang et al., 2014; de Bruijn et al., 2015; Liu et al., 2015). Despite all this structural and functional information, characterisation of the mechanisms involved in the motility of *Lysobacter* cells is still very poor.

Recently, Zhou et al. (2015) observed that *L. enzymogenes* C3 cells located at the edge of the colonies were able to move, possibly using some sort of twitching motility. Some *L. capsici* and *L. enzymogenes* strains were reported to be able to disperse on agar surface after 12 days of incubation, and this movement was associated with gliding motility (Gómez Expósito et al., 2015). Similarly, we observed that dispersal of *L. capsici* AZ78, a biocontrol agent of *Phytophthora infestans* and *Plasmopara viticola* (Puopolo et al., 2014a,b), occurred when the bacterium was grown on a medium containing pea broth used in a dual-culture assay with *P. infestans* (**Supplementary Figure S1**).

Based on this observation, trials were carried out under greenhouse conditions to assess the contribution of the motility due to the pea broth in the biocontrol activity of *L. capsici* AZ78 against *P. viticola*. Subsequently, we investigated the ability of several strains belonging to various *Lysobacter* species to move on inert surfaces through swimming, swarming and twitching motility and we observed specific medium-dependent motility in several *L. capsici* strains. The availability of the *L. capsici* AZ78 genome (Puopolo et al., 2016) made it possible to identify genes encoding proteins involved in the flagellum and T4P biogenesis. Furthermore, relative gene expression analysis revealed that *L. capsici* AZ78 motility on pea broth jellified medium is associated with the upregulation of genes related to T4P machinery.

## Materials and Methods

### Bacterial Strains and Growth Media

The bacterial strains used in this study (**Table 1**) were stored in 40% glycerol at -80°C and routinely grown on Luria Bertani Agar [LBA; LB broth (Sigma Chemical-St. Louis, MO, USA), 1.6% (w/v) Agar Technical No.3 (Agar, Oxoid-Columbia, MD, USA)] at 27°C. Bacterial cultures originating from 72 h incubation at 27°C were used in all the experiments, unless otherwise indicated.

**Table 1:** Bacterial strains.

Species	Strain	Origin	Reference
*Lysobacter antibioticus*	DSM 2044^T^	Soil	DSMZ
*Lysobacter arseniciresistens*	DSM 2723^T^	Soil	DSMZ
*Lysobacter brunescens*	DSM 6979^T^	Lake water	DSMZ
*Lysobacter capsici*	DSM 19286^T^	Pepper rhizosphere	DSMZ
*Lysobacter capsici*	DSM 23109^T^	Clay soil (grass crop)	DSMZ
*Lysobacter capsici*	AZ78	Tobacco rhizosphere	Puopolo et al., 2014b
*Lysobacter capsici*	M143	Tomato rhizosphere	Puopolo et al., 2014b
*Lysobacter capsici*	55	Clay soil (cauliflower crop)	Postma et al., 2010
*Lysobacter capsici*	6.2.3	Clay soil (grass crop)	Postma et al., 2010
*Lysobacter daejeonensis*	DSM 17634^T^	Greenhouse soil	DSMZ
*Lysobacter enzymogenes*	DSM 2043^T^	Soil	DSMZ
*Lysobacter gummosus*	DSM 6980^T^	Soil	DSMZ
*Lysobacter spongiicola*	DSM 21744^T^	Deep-sea sponge	DSMZ
*Bacillus amyloliquefaciens*	S499	Soil	Pertot et al., 2013
*Pseudomonas chlororaphis*	M71	Tomato rhizosphere	Puopolo et al., 2011


*^T^Type strain; DSMZ, Leibniz Institute DSMZ-German Collection of Microorganism and Cell Cultures.*

Agar (Oxoid) was added at different concentrations in all the growth media used. Swimming Agar [SWM; 1.0% Tryptone (Oxoid), 0.5% NaCl (Sigma–Aldrich), 0.3% (w/v) Agar, pH 7.00] and Swarming Agar [SWR, 0.8% Nutrient broth (NB) No.2 (Fluka analytical), 0.5% D-(+)-Glucose (Sigma–Aldrich), 0.5% (w/v) Agar] were used for swimming and swarming motility assays, respectively, (Rashid and Komberg, 2000; Déziel et al., 2001). LB amended with 0.5% (LBA 0.5) and 1% Agar (w/v) (LBA 1) were used for the swarming and twitching motility assays, respectively, (Rashid and Komberg, 2000; Dunger et al., 2014). Pea broth (PB; 12.5% frozen peas in distilled water) amended with 0.3% [Pea Agar Medium (PAM) 0.3]; 0.5% (PAM 0.5) and 1% Agar (w/v) (PAM 1) were used for the swimming, swarming and twitching motility assays, respectively.

### Analysis of Pea Broth Effects on the *In vivo* Activity of *Lysobacter capsici* AZ78

The PB effect on the efficacy of *L. capsici* AZ78 against *P. viticola* under controlled greenhouse conditions was tested according to Puopolo et al. (2014b). Two-year-old *Vitis vinifera* cv. Pinot Noir grapevine plants, grafted onto Kober 5BB, were treated with distilled water (H_2_O), PB, PB augmented with *L. capsici* AZ78 (1 × 10^8^ cells/ml) or *L. capsici* AZ78 (1 × 10^8^ cells/ml). Each treatment (40 ml/plant) was applied on adaxial and abaxial leaf surfaces 24 h before inoculation with *P. viticola* (2.5 × 10^5^ sporangia/ml). Both treatments and inoculum were sprayed with a hand sprayer. Inoculated plants were incubated overnight at 25 ± 1°C and 80–99% Relative Humidity (RH) in the dark, and then maintained at 25 ± 1°C and 60–80% RH with a 16/8-h day/night light regime. Seven days after inoculation, plants were incubated overnight in the dark at 25 ± 1°C and 80–99% RH to induce *P. viticola* sporulation. Disease severity (percentage of abaxial leaf area covered with sporulating lesions) and disease incidence (percentage of leaves with visible sporulation) were evaluated visually according to the standard guidelines of the European and Mediterranean Plant Protection Organization (EPPO, 2001). A randomized complete block design (six plants per treatment) was used. The presence of *L. capsici* AZ78 cells on grapevine leaves was assessed 24 h after the treatment with the dilution plating method (Puopolo et al., 2014b).

### Motility Assays on Inert Surfaces

The motility of the *Lysobacter* strains (**Table 1**) was evaluated on different media. The experiments to assess swimming and swarming motility were carried out according to Köhler et al. (2000), with modifications. Briefly, 18 ml of each medium (SWM, PAM 0.3, SWR, LBA 0.5, and PAM 0.5) were poured into Petri dishes (90 mm) and dried for 1 h on the bench at room temperature and 20 min under laminar flow. Once dry, the dishes were inoculated with each bacterial strain with a toothpick and maintained at 27°C for 20 h.

The twitching motility assay was carried out according to Dunger et al. (2014), with modifications. Briefly, the bacterial strains were inoculated with a toothpick at the bottom of the dishes containing LBA 1 and PAM 1. After 7 days of incubation at 27°C, the agar was removed and the zone of twitching motility was dyed with 0.1% (w/v) crystal violet (CV) at room temperature for 15 min. *Bacillus amyloliquefaciens* S499 (Pertot et al., 2013) and *Pseudomonas chlororaphis* M71 (Puopolo et al., 2011) were used as a control in all the assays. For each motility assay, three Petri dishes of each medium were used for each bacterial strain.

To evaluate whether PB may foster *L. capsici* AZ78 motility *in planta*, autoclaved PB (200 ml) cooled down to room temperature was applied with a hand sprayer to the leaves of three grapevine plants (replicates) and left to dry for 1 h under greenhouse conditions. Grapevine plants treated with 200 ml of distilled water (H_2_O) were used as a control. Subsequently, ten leaves from each grapevine plant were collected and placed in sterile plastic boxes containing 100 ml of double distilled water and washed for 1 h with orbital shaking (100 rpm) at room temperature. The resulting leaf-washing suspensions were filtered with sterile cheesecloth and collected in sterile bottles. Agar was added to each leaf-washing solution to reach a final concentration of 0.5% (w/v) and subsequently autoclaved. The resulting media were poured into Petri dishes (18 ml each) and dried as described previously. *L. capsici* AZ78 was inoculated with a toothpick and colony areas were measured after 20 h of incubation at 27°C as described above. Three Petri dishes were used for each replicate.

For each assay, Petri dishes were visualized with a Bio-Rad Geldoc system and images were digitally captured using Bio-Rad Quantity One software. The motility of bacterial strains on inert surfaces was subsequently quantified by scoring the colony areas (mm^2^) using Fiji software (ImageJ 1.49; Dunger et al., 2014).

### Genome Mining

The sequenced genome of *L. capsici* AZ78 (version JAJA00000000.2; Puopolo et al., 2016) was mined to identify putative genes involved in bacterial motility mechanisms using nucleotide and protein sequence comparison. To identify putative *L. capsici* AZ78 genes responsible for flagellum and T4P, annotated products of genes encoding regulatory and structural components of flagella and T4P of *L. capsici* 55 (CP011130), *P. aeruginosa* PAO1 (AE004091), *Stenotrophomonas maltophilia* (*Sm*) K279a (AM743169), and *Xanthomonas campestris* (*Xc*) pv. *campestris* ATCC 33913 (AE008922; Stover et al., 2000; da Silva et al., 2002; Crossman et al., 2008; de Bruijn et al., 2015) were aligned against the *L. capsici* AZ78 genome, using the RAST server (Aziz et al., 2008), and a cut-off of 1e-10 at amino acid level was applied. The putative *L. capsici* AZ78 genes identified were then analyzed with BLATP (Johnson et al., 2008), and length >70 and identity ≥50% at amino acid level were used as thresholds.

Primers specific (**Supplementary Table S1**) for flagellum and T4P biogenesis in *L. capsici* AZ78 were designed using Primer3 software^1^ (Untergasser et al., 2012) and their specificity was assessed through PCR and Sanger sequencing before gene expression analysis using quantitative real-time polymerase chain reaction (qRT-PCR).

### Assessment of *Lysobacter capsici* Az78 Cell Growth in Different Media

The influence of the medium on the cell growth of *L. capsici* AZ78 was assessed in LB, PB, and SWR broth [NB; 0.5% D-(+)-glucose (Sigma–Aldrich) (w/v)]. Cell growth was measured as optical density at 600 nm (OD_600_) using a Synergy 2 agitated multiwell plate reader (Biotek, Winooski, VT, USA) according to Segarra et al. (2015). The OD_600_ was monitored each hour for 36 h at 27°C. The initial bacterial cell concentration was 1 × 10^7^ cells/ml (Puopolo et al., 2014b). Non-inoculated media were used as a control. Seven technical replicates were carried out.

### Gene Expression Analysis

*Lysobacter capsici* AZ78 was inoculated on LBA 0.5, PAM 0.5, and SWR with a toothpick and afterward incubated at 27°C for 20 h. Then plugs (5-mm diameter) were collected from the bacterial macrocolony, immediately frozen in liquid nitrogen and ground to a fine powder. Three Petri dishes for each medium were inoculated (replicates). Each replicate was composed of three plugs from *L. capsici* AZ78 macrocolonies originating from the same dish.

Total RNA was extracted with Tri Reagent (Sigma–Aldrich, St. Louis, MO, USA), according to the manufacturer’s instructions, with slight modifications. The final pellet was re-suspended in 50 μl of RNase-free water. Total RNA was subsequently purified using the RNAeasy Mini Kit (Qiagen Sciences, Valencia, CA, USA) and DNase treatment was performed with the RNase-Free DNase set (Qiagen). RNA integrity and concentration were assessed using electrophoresis in agarose gel and a Qubit 3.0 Fluorometer (Invitrogen), respectively. First-strand cDNA was synthesized from 100 ng of purified RNA with the SuperScript III Reverse Transcriptase RT kit (Invitrogen), according to the manufacturer’s instructions. All qRT-PCR reactions were carried out with Platinum SYBR Green qPCR SuperMix-UDG (Invitrogen) and specific primers (**Supplementary Table S1**) using LightCycler 480 software (Roche Diagnostics, Mannheim, Germany). The qRT-PCR reactions consisted of 50 amplification cycles (95°C for 15 s and 60°C for 45 s) and melting curve analysis. Cycle threshold (Ct) values were extracted with LightCycler 480 SV1.5.0 software (Roche Diagnostics) using the second derivative calculation and reaction efficiency was calculated with LinRegPCR 11.1 software (Ruijter et al., 2009). Relative expression levels were determined with the 2^-ΔΔCt^ method (Pfaffl, 2001) based on three replicates per sample using *L. capsici* AZ78 growth on LBA 0.5 as the calibrator. The housekeeping gene *recA* (AZ78_1089) was used as the constitutive gene for normalization, because its expression was not significantly affected by growth media and conditions (Takle et al., 2007; Florindo et al., 2012). qRT-PCR reactions were carried out for the two independent experiments.

### Influence of Pea Concentrations on the Motility of *Lysobacter capsici* AZ78

The influence of nutrient concentration on *L. capsici* AZ78 motility was evaluated using PAM with 0.5% Agar (w/v) and frozen peas at four concentrations: 1.5, 3.0, 6.0, and 12.5% (w/v). Swarming motility experiments were carried out as described above. Three Petri dishes were used as replicates.

### Transmission Electron Microscopy

Drops (50 μl) containing a suspension of *L. capsici* AZ78 cells (1 × 10^8^ cells/ml), following 20 h incubation at 27°C on PAM 0.5 and LBA 0.5, were adsorbed to transmission electron microscopy (TEM) carbon-formvar coated nickel grids for 10 min, at room temperature. The bacterial cells were then stained in 3% (w/v) uranyl-acetate for 8 min and rinsed three times (10 s each) in sterile distilled water. The grids were examined under a Philips CM 10 TEM (Eindhoven, The Netherlands) operating at 80 kV (Cowles and Gitai, 2010).

### Statistical Analysis

Swarming, swimming and twitching assays and the experiments regarding the influence of pea concentration on the *L. capsici* AZ78 motility were carried out three times while the remaining experiments were carried out twice. All statistical tests were carried out using Statistica 9.0 (StatSoft, USA). For each assay the data obtained from the repeated experiments were subjected to two-way analysis of variance (ANOVA) and data were pooled when no significant differences were found, according to the *F*-test (α > 0.05). Data on disease incidence and severity were log transformed, while the fold change values of gene expression analysis were transformed using the equation y = log_10_(1+x) (Casagrande et al., 2011). The data for swarming, qRT-PCR, disease severity, disease incidence and cell density were analyzed using one-way ANOVA, after validation of normal distribution (*K–S* test, α > 0.05) and variance homogeneity of the data (Levene’s test, α > 0.05), Tukey’s test (α = 0.05) was applied to detect significant differences. Student’s *t*-test (α = 0.05) was applied in pairwise comparison of the colony areas reached in the swimming, twitching, and *in planta* motility assays.

## Results

### Pea Broth Enhances the Plant Protection Efficacy of *Lysobacter capsici* AZ78

Based on the occurrence of motility observed when *L. capsici* AZ78 was grown on jellified PB (**Supplementary Figure S1**), greenhouse trials were set up to assess the impact of PB on the efficacy of *L. capsici* AZ78 in controlling *P. viticola.* Although an effect of the experiment was present (*F*-test, *P* = 0.004), the application of *L. capsici* AZ78 alone and *L. capsici* AZ78 with PB significantly reduced disease severity and incidence, as compared with H_2_O and PB-treated plants (**Table 2**). In particular, disease severity and incidence were significantly lower in plants treated with *L. capsici* AZ78 and PB than with *L. capsici* AZ78 alone. As expected, no *L. capsici* AZ78 cells were isolated from leaves of H_2_O and PB-treated plants. *L. capsici* AZ78 cells recovered from leaves treated with *L. capsici* AZ78 alone were lower than those recovered from leaves treated with *L. capsici* AZ78 and PB (**Table 2**).

**Table 2:** Effect of pea broth on the plant protection efficacy of *Lysobacter capsici* AZ78.

Experiment 1	Disease Severity (%)	Disease Incidence (%)	Cell Density (log_10_ CFU g^-1^ of leaf)
H_2_O	24.7 ± 2.3^a^	100 ± 0^a^	0 ± 0^c^
PB	21.7 ± 4.2^a^	100 ± 0^a^	0 ± 0^c^
PB + *L. capsici* AZ78	3.4 ± 0.8^c^	47.9 ± 6.2^c^	5.5 ± 0.3^a^
*L. capsici* AZ78	7.9 ± 1.4^b^	87.0 ± 1.4^b^	4.2 ±0.1^b^

**Experiment 2**	**Disease Severity (%)**	**Disease Incidence (%)**	**Cell Density (log_10_ CFU g^-1^ of leaf)**

H_2_O	29.3 ± 5.0^a^	100 ± 0^a^	0 ± 0^c^
PB	30.2 ± 4.4^a^	100 ± 0^a^	0 ± 0^c^
PB + *L. capsici* AZ78	0.8 ± 0.2^c^	10.7 ± 2.7^c^	6.2 ± 0.1^a^
*L. capsici* AZ78	2.5 ± 0.3^b^	43.1 ± 5.2^b^	5.3 ± 0.1^b^


*The treatments applied to grapevine plants were: distilled water (H_2_O), pea broth (PB), *L. capsici* AZ78 (1 × 10^*8*^ cells/ml) and the combination of pea broth and *L. capsici* AZ78 (1 × 10^*8*^ cells/ml). Disease severity (% of abaxial leaf area covered with sporulating lesions) and disease incidence (% of leaves with visible sporulation) were evaluated seven days after *P. viticola* inoculation. The density of *L. capsici* AZ78 cells residing on grapevine leaves was evaluated using a dilution plating method. Two separate trials (Experiment 1 and Experiment 2; [F-test; *P* = 0.004]) were carried out and six plants (replicates) were used for each treatment. Mean ± standard error values are reported in the table. Different letters indicate significant differences according to Tukey’s test (α = 0.05).*

### The Motility of *Lysobacter capsici* Depends on Medium Composition

**FIGURE 1:**
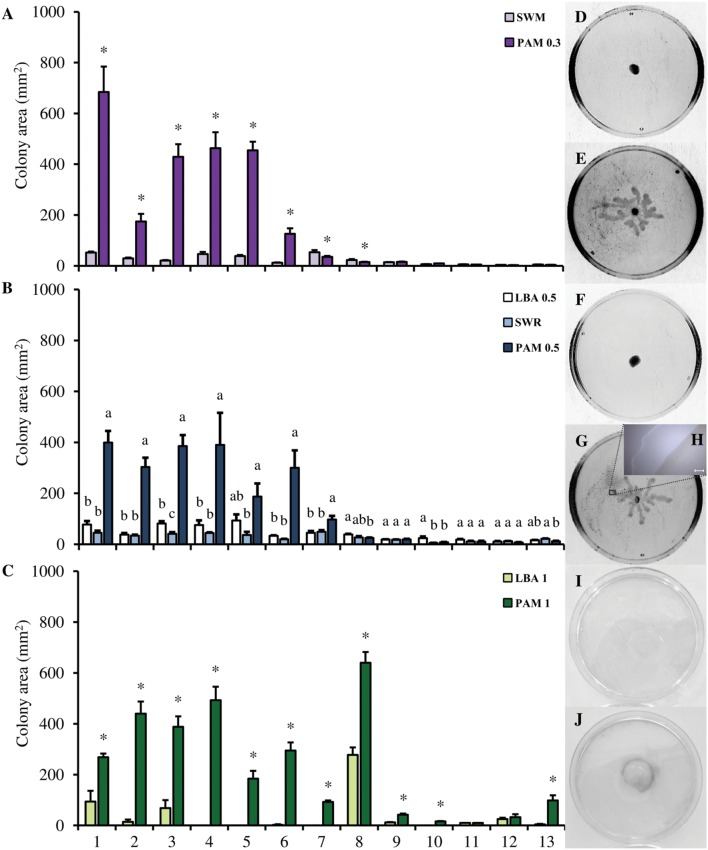
**Motility of the *Lysobacter* species on inert surfaces.** The swimming **(A)**, swarming **(B)** and twitching **(C)** motility of the *Lysobacter* species (colony area, mm^2^) was evaluated on Swimming Agar, (SWM), Pea Agar Medium 0.3 (PAM 0.3), Luria Bertani Agar 0.5 (LBA 0.5), Swarming agar (SWR), Pea Agar Medium 0.5 (PAM 0.5), Luria Bertani Agar 1 (LBA 1), and Pea Agar Medium 1 (PAM 1). An *F*-test revealed non-significant differences between experiments (*P* values ranged from 0.12 to 0.81 for the motility assay) and data from the three experiments were pooled. Mean colony area and standard error values were calculated as the pool of nine replicates (Petri dishes) from three experiments. For each strain, asterisks indicate values that differ significantly according to Student’s *t*-test (α = 0.05) in the pairwise comparison of SWM and PAM 0.3, and LBA 1 against PAM 1. In the swarming assay for each strain, different letters indicate significant differences according to Tukey’s test (α = 0.05). 1, *L. capsici* AZ78; 2, *L. capsici* 55; 3, *L. capsici* 6.2.3.; 4, *L. capsici* DSM 19286^T^; 5, *L. capsici* DSM 23109; 6, *L. capsici* M143; 7, *L. antibioticus* DSM 2044^T^; 8, *L. enzymogenes* DSM 2043^T^; 9, *L. daejeonensis* DSM 17634^T^; 10, *L. gummosus* DSM 6980^T^; 11, *L. brunescens* DSM 6979^T^; 12, *L. spongiicola* DSM 21744^T^; 13, *L. arseniciresistens* DSM 2723^T^. Pictures of *L. capsici* AZ78 motility on SWM **(D)**, PAM 0.3 **(E)**, LBA 0.5 **(F)**, PAM 0.5 **(G)**, LBA 1 **(I)**, and PAM 1 **(J)**. The images were captured using Bio-Rad Quantity One software. The detail of the biosurfactant ring **(H)** was taken with a LMD7000 instrument (Leica Microsystems), software LAS V3.7 (Leica Microsystems); Magnification X-5, bar 200 μm.

*Bacillus amyloliquefaciens* S499 and *P. chlororaphis* M71, used as controls, were able to move on all the media employed in the motility assays (**Supplementary Table S2**). No significant differences were found between the swimming motility on SWM and PAM 0.3 in type strains of *L. antibioticus, L. arseniciresistens, L. brunescens, L. daejeonensis, L. enzymogenes, L. gummosus*, and *L. spongiicola* (**Figure 1A**). The colony area of *L. capsici* strains grown on PAM 0.3 [ranging from 125.8 ± 22.0 mm^2^ of *L. capsici* M143 (mean ± SE) to 684 ± 99.7 mm^2^ of *L. capsici* AZ78], was significantly larger than the area measured on SWM [ranging from 12.5 ± 0.8 of *L. capsici* M143 to 52.2 ± 3.7 mm^2^ of *L. capsici* AZ78; **Figures 1A,D,E**]. However, the colony morphology of *L. capsici* strains grown on PAM 0.3 did not show the typical swimming morphology registered in the case of *B. amyloliquefaciens* S499 and *P. chlororaphis* M71 (**Supplementary Figure S2**). In particular, the colony morphology of all *L. capsici* strains on PAM 0.3 was characterized by the presence of multiple dendrites originating from the inoculation spot and elongating toward the edge of the Petri dish (**Figure 1E**).

*Lysobacter antibioticus, L. arseniciresistens, L. brunescens, L. daejeonensis, L. enzymogenes, L. gummosus*, and *L. spongiicola* type strains did not swarm on LBA 0.5, PAM 0.5, and SWR. They developed a circular macrocolony and the growth area was limited to the inoculation spot on the three media (**Figures 1B,F,G**). All the *L. capsici* strains showed swarming motility and spread on PAM 0.5, forming dendrites as in the swimming test (**Figure 1G**). Moreover, significant differences between the areas observed on PAM 0.5 and the two other media were observed (**Figure 1B**). For example, *L. capsici* AZ78 grown on PAM 0.5 had a colony area of 399.1 ± 45.7 mm^2^, significantly larger than the area covered on LBA 0.5 (45.0 ± 8.7 mm^2^) and SWR (77.5 ± 13.5 mm^2^). Interestingly, a ring halo caused by the production of biosurfactant compounds was observed in the case of *L. capsici* strains grown on PAM after 20 h of incubation at 27°C (**Figure 1H**).

When LBA 1 was used, *L. antibioticus*, *L. arseniciresistens*, *L. brunescens*, *L. capsici*, *L. daejeonensis*, *L. gummosus*, and *L. spongiicola* strains did not produce a visible twitching zone. *L. enzymogenes* DSM 2043^T^ was the only strain that developed a visible twitching zone, with an area of 277.4 ± 29.2 mm^2^ (**Figures 1C,I,J**). However, *L. antibioticus*, *L. arseniciresistens*, *L. daejeonensis*, *L. enzymogenes*, *L. gummosus*, and all the *L. capsici s*trains developed a visible twitching zone on the plastic surface when PAM 1 was used. On this medium, the largest areas were developed by *L. enzymogenes* DSM 2043^T^ (639.6 ± 42.7 mm^2^) and the *L. capsici* strains (ranging from 184.0 ± 30.9 mm^2^ of *L. capsici* DSM 23109 to 9388.1 ± 41.1 mm^2^ of *L. capsici* 6.2.3) (**Figure 1J**).

**FIGURE 2:**
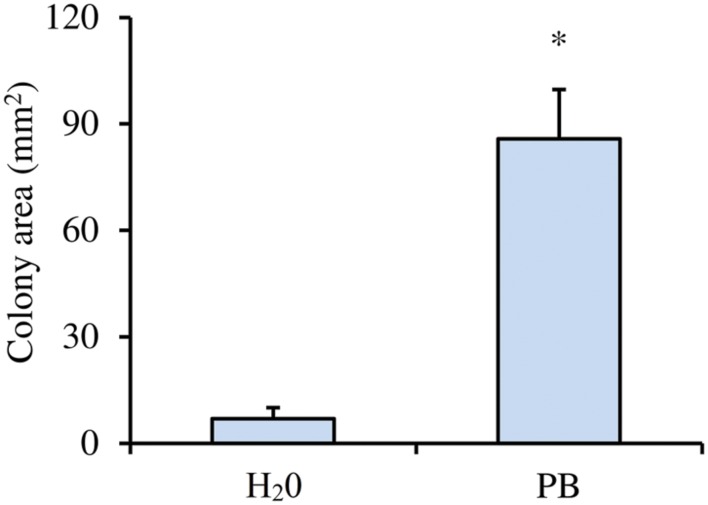
**Effect of pea broth deposited on grapevine leaves on *in vitro* motility of *Lysobacter capsici* AZ78.** The bacterial colony area (mm^2^) of *L. capsici* AZ78 was measured on media (0.5% Agar) coming from the leaf-washing suspension of grapevines treated with pea broth (PB) and distilled water (H_2_O). An *F*-test revealed non-significant differences between experiments (*P* = 0.73), and data from the two experiments were pooled. Mean and standard error values were calculated as the pool of three replicates from two experiments. Asterisks indicate values that differ significantly according to Student’s *t*-test (α = 0.05).

Moreover, leaf-washing suspensions deriving from plants treated with PB allowed significantly greater motility of *L. capsici* AZ78 cells, as compared with those deriving from plants treated with H_2_O (**Figure 2**).

### The Genome of *Lysobacter capsici* Az78 Includes Genes Responsible for Flagellum and Type IV Pilus Biogenesis

The flagellar gene organization of *L. capsici* AZ78 matched that of *L. capsici* 55, *L. enzymogenes* C3, *L. enzymogenes* OH11, *L. gummosus* 3.2.11, and the oceanic γ-Proteobacteria *Haella chejuensis* KCTC 2396^T^ (CP000155; Jeong et al., 2005; Wang et al., 2014; de Bruijn et al., 2015). In particular, 21 putative genes encoding components of flagellar apparatus are present in the *L. capsici* AZ78 genome (**Supplementary Table S3**). These genes are highly conserved in *L. capsici* 55 (de Bruijn et al., 2015) and members of the two phylogenetically related flagellated species *Xc* and *Sm.* For instance, the amino acid sequence of the basal body components *flgI* and *flgH* (AZ78_1627, 1628) are 100 and 99% identical with the flagellar P-ring protein and L-ring protein of *L. capsici* 55 (ALN84757, ALN84756), respectively. The same amino acid sequences are 75 and 71% identical with the basal body proteins of *Sm* (KOO80985, KOO78078). The putative *flgE* (AZ78_1616) shared 99 and 60% of identity at amino acid level with the flagellar hook protein of *L. capsici* 55 (ALN84768) and *Sm* (KOO78088), respectively. Through BLASTP analysis, a gene (AZ78_1611) including two sigma-70 conserved regions was identified in the *L. capsici* AZ78 genome. This transcriptional factor was 99 and 39% identical with the RNA polymerase sigma-70 factor of *L. capsici* 55 (ALN84773) and *L. enzymogenes* C3 (ALN59990), respectively. No ortholog of *fliC* was found in the genome of *L. capsici* AZ78.

Genome mining allowed identification of 43 genes related to T4P biogenesis, and they were organized into six main gene clusters (**Supplementary Table S4**), as reported for other *Lysobacter* strains (de Bruijn et al., 2015). The putative T4P genes of *L. capsici* AZ78 had high sequence identity with the orthologs found in members of the *Xc* and *Sm* species. The genome of *L. capsici* AZ78 had two genes encoding type IV major pilin PilA (AZ78_4276, 4277), and their amino acid sequence identity ranged from 42 to 56% with the *Xc* ortholog (KOB01592) and 31 to 73% with the protein products of *xac*3240–3241 of *X. axonopodis* pv. *citri* 306 (AE008923). Moreover, the PilA proteins (AZ78_4276, 4277) shared 59 and 68% amino acid identity with the pilin ortholog of *L. capsici 55* (ALN87506). Interestingly, the *L. capsici* AZ78 genome included a third putative pilin gene (AZ78_3612) that was 39% identical with XAC3805 of *X. axonopodis* pv. *citri* 306 at amino acid level, and it was located far from the *pilRS* (5,121,276-5,124,387 bp) and *pilABCD* (5,105,118-5,120,729 bp) regions.

**FIGURE 3:**
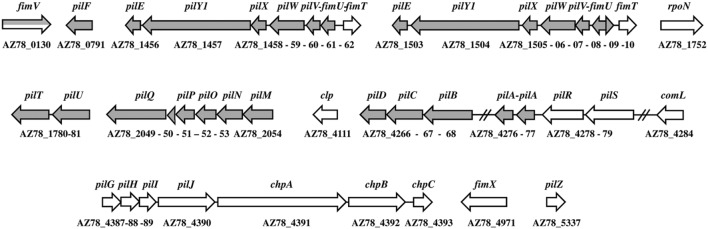
**Type IV pili gene organization in the *Lysobacter capsici* AZ78 genome.** Putative genes encoding structural components of the type IV pilus are in gray, and putative genes encoding regulatory components are in white. The gene names are given above the arrows. The corresponding accession number is given under each gene.

The minor pilin operon (*fimUpilVWXY1E*) was repeated in the genome of *L. capsici* AZ78 (1,769,261–1,776,246 and 1,814,313–1,821,233 bp; **Figure 3**), similarly to *L. capsici* 55, *L. enzymogenes* C3, and *L. gummosus* 3.2.11 (de Bruijn et al., 2015). The genes were highly conserved at amino acid level through *Lysobacter* members with an identity value ranging from 59 to 100% (**Supplementary Table S4**). The *L. capsici* AZ78 pilus-specific chemotaxis system (Pil-Chp) was composed of seven *pilGHIJchpABC* genes (5,263,810–5,277,356 bp) and was missing a *pilK* ortholog. Interestingly, the putative assembly protein PilG (AZ78_4387) shared 86% identity with the *P. aeruginosa* ortholog (KSF29090) and more than 90% with the corresponding orthologs in *L. enzymogenes* C3 (ALN59304; Zhou et al., 2015), *Xc* (KOB02299) and *Sm* (KIP84116). The alignment of the subcomplex operon *pilMNOP* (2,509,989–2,513,122 bp) and *pilQ* (AZ78_2049) revealed that these genes were highly conserved through the *Lysobacter* members (from 83 to 100% amino acid identity) and the genes shared from 61 to 82% of amino acid sequence identity with *Xc* and *Sm*, respectively.

The T4P transcriptional factor RpoN (𝔖^54^) (AZ78_1752) was 67 and 65% identical to the ortholog protein of *Xc* (CDN18987) and *Sm* (KOQ68432), respectively. The amino acid identity of this transcriptional factor ranged from 88 to 100% through the *Lysobacter* species (e.g., *Lysobacter antibioticus* ATCC 29479, ALN61594; *L. capsici* 55, ALN84614). Moreover, the two-component system PilR–PilS, expressed by the gene operon AZ78_4278-4279, showed high amino acid identity (from 58 to 73%) with the PilR-PilS proteins of *Xc* (NP_638443-NP_638442) and *Sm* (WP_049405671-WP_017356171). The *pilR–pilS* operon was highly conserved in *L. capsici* AZ78 and *L. capsici* 55 (ALN87505, ALN87504), indeed the protein sequences were 100 and 99% identical, respectively. The derived amino acid sequence of the global regulator *clp* (AZ78_4111) was 84 and 45% identical to the ortholog Clp of *Xc* (NP_635866) and Vfr of *P. aeruginosa* PAO1 (NP_249343), respectively, (Beatson et al., 2002; He et al., 2007; Burrows, 2012). The Clp amino acid sequence was conserved in *Lysobacter* members with an identity value of 99% (e.g., *L. capsici* 55, ALN87652; *L. enzymogenes* C3, AAP83141).

### Upregulation of Genes Involved in Type IV Pilus Biogenesis is Associated with *Lysobacter* Az78 Medium-Dependent Motility

*Lysobacter capsici* AZ78 grew in a similar way in LB, PB and SWR broth (**Supplementary Figure S3**). The bacterial strain entered the logarithmic phase after 6 h in all the media. However, the cell mass produced in PB was lower than LB and SWR (**Supplementary Figure S3**).

**FIGURE 4:**
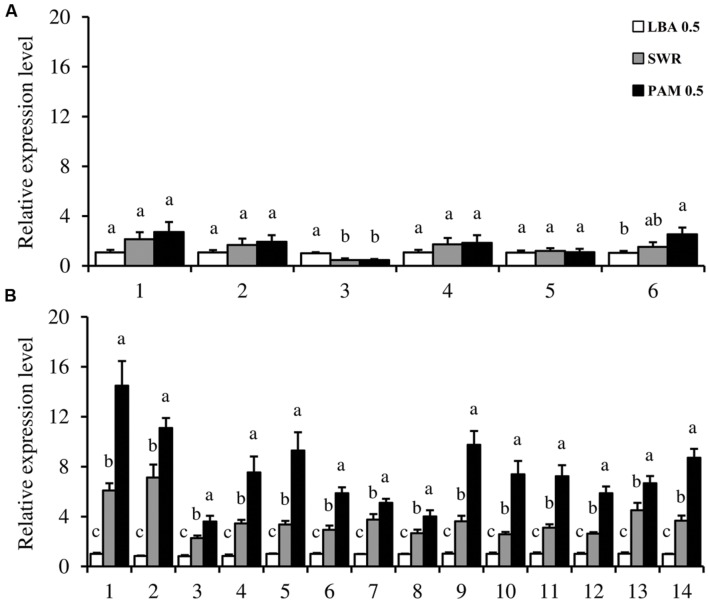
**Expression analysis of flagella and type IV pili-related genes in *Lysobacter capsici* AZ78.** The expression of genes encoding flagellar **(A)** and type IV pili **(B)** regulatory and structural proteins was analyzed when *L. capsici* AZ78 was grown for 20 h on Luria Bertani Agar (LBA 0.5), Swarming Agar (SWR) and Pea Agar Medium (PAM 0.5); the media were amended with 0.5% Agar (w/v). Relative expression levels were calculated using *recA* as the constitutive gene for data normalization, and data were calibrated on *L. capsici* AZ78 growth on LBA 0.5 samples. An *F*-test revealed non-significant differences between experiments (*P* values ranged from 0.08 to 0.96, for the genes tested), and data from the two experiments were pooled. Mean and standard error values were calculated as the pool of six replicates from two experiments. For each gene, different letters indicate significant differences according to Tukey’s test (α = 0.05). **(A)** Flagellar genes: 1, *flhB*; 2, *fliR*; 3, *flgE*; 4, *flgI*; 5, *fliH*; 6, *fliA.* **(B)** T4P genes: 1, *pilA*.1; 2, *pilA*.2; 3, *pilZ*; 4, *pilY*1; 5, *pilB*; 6, *clp*; 7, *fimX*; 8, *rpoN*; 9, *pilM*; 10 *pilG*; 11, *pilI*; 12, *pilL*; 13, *pilJ*; 14, *pilQ*.

Six and 14 genes, respectively, responsible for flagellum and T4P biogenesis in *L. capsici* AZ78 were selected for gene expression analysis using qRT-PCR (**Supplementary Table S1**). The relative expression levels of the structural and regulatory components of flagellum and T4P were calculated for *L. capsici* AZ78 cells grown on LBA 0.5, PAM 0.5, and SWR. qRT-PCR analysis revealed the absence of transcriptional regulation of the structural flagellar genes *flgI* (AZ78_1627), *flhB* (AZ78_1633), *fliH* (AZ78_1621), and *fliR* (AZ78_1634) in the three media tested (**Figure 4A**). The expression of putative *flgE* (AZ78_1616) was down regulated (0.5-fold) when *L. capsici* AZ78 was grown on both PAM 0.5 and SWR, compared with LBA 0.5 (**Figure 4A**). The putative RNA-polymerase 𝔖^70^ regulatory factor (*fliA*, AZ78_1611) was induced more than twofold on PAM 0.5 compared with LBA 0.5, but the gene expression level on PAM 0.5 was comparable to that on SWR (**Figure 4A**).

Gene expression analysis revealed significant upregulation of all the T4P biogenesis genes when *L. capsici* AZ78 was grown on PAM 0.5, compared with LBA 0.5 and SWR (**Figure 4B**). The gene expression levels of the four apparatus sub-complexes were induced by growth on PAM 0.5 medium as compared with SWR and LBA 0.5. In particular, the putative *pilQ* (AZ78_2049) and *pilM* (AZ78_2054) genes were upregulated on PAM 0.5 (more than ninefold) and on SWR (more than fourfold), compared with LBA 0.5. The expression level of the putative *pilB* (AZ78_4268) on PAM 0.5 was nine and threefold higher than on LBA 0.5 and SWR, respectively. The putative *pilY1* (AZ78_1457) was upregulated on PAM 0.5 (ninefold) and SWR (fourfold), compared with LBA 0.5. The major pilin genes *pilA* (AZ78_4276, 4277) were induced more than 13-fold when *L. capsici* AZ78 was grown on PAM 05, as compared to LBA 0.5 (**Figure 4B**). Moreover, the expression levels of *pilA* genes on PAM 0.5 were twice as high as those on SWR (sevenfold). As regards the regulatory system, the putative *pilJ* (AZ78_4390), encoding the single methyl-accepting chemotaxis protein (MCP) of the Pil-Chp system, was positively regulated when the bacterium was grown on PAM 0.5 (6.5-fold) and SWR (1.5-fold), as compared with LBA 0.5. Moreover, the other components of the Pil-Chp system tested [*pilG* (AZ78_4387), *pilI* (AZ78_4389), and *chpA* (AZ78_4391)] were upregulated on PAM 0.5 (five to sevenfold) and SWR (two to threefold), as compared with LBA 0.5. The gene encoding the regulatory factor RpoN (𝔖^54^) (AZ78_1752) was induced (fourfold) on PAM 0.5, as compared to LBA 0.5. In addition, growth on PAM 0.5 increased the *clp* (AZ78_4111) expression level sixfold as compared with LBA 0.5.

**FIGURE 5:**
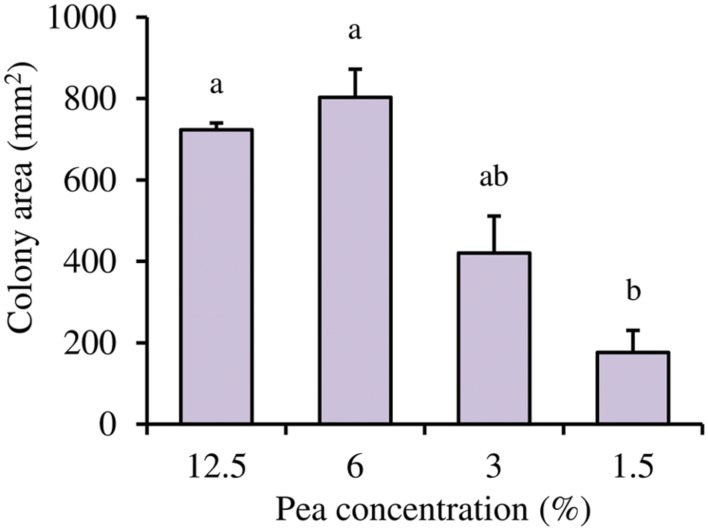
**Influence of pea concentrations on *Lysobacter capsici* AZ78 motility.** The motility of *Lysobacter capsici* AZ78 was monitored on Pea Agar Medium (0.5% Agar) containing 12.5, 6.0, 3.0, and 1.5% frozen peas. An *F*-test revealed non-significant differences between experiments (*P* = 0.84), and data from the three experiments were pooled. Mean colony area (mm^2^) and standard error values were calculated as the pool of nine replicates (Petri dishes) from three experiments. Different letters indicate significant differences according to Tukey’s test (α = 0.05).

Based on the qRT-PCR analysis, the possible implication of the pea concentration on *L. capsici* AZ78 motility was assessed. *L. capsici* AZ78 bacterial motility was negatively influenced by low nutrient availability. Indeed, a reduction in the *L. capsici* AZ78 colony area was registered in the assays, with a significant difference between the highest (12.5%) and lowest (1.5%) pea concentration (from 723.4 ± 16.4 mm^2^ to 175.7 ± 54.9 mm^2^; **Figure 5**).

**FIGURE 6:**
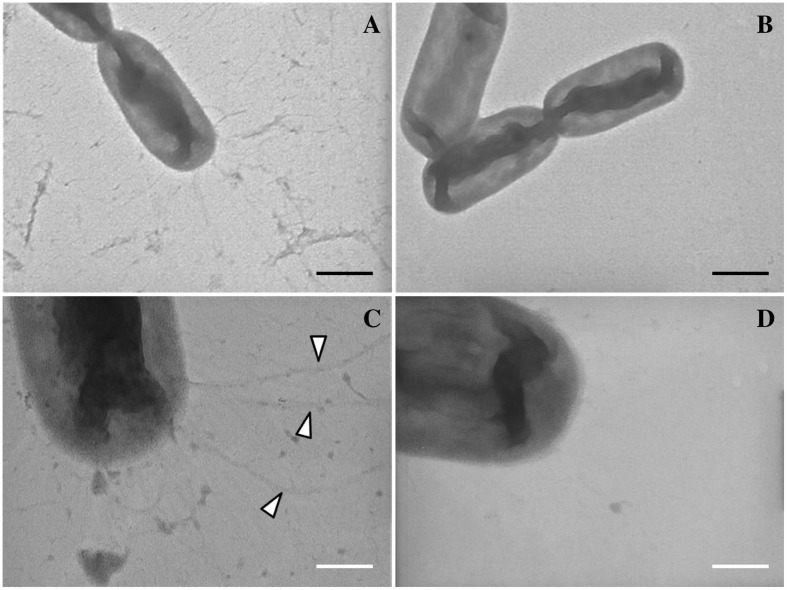
**Visualization of type IV pili of *Lysobacter capsici* AZ78 using Transmission Electron Microscopy (TEM).** Pilus-like structures (arrows) emerge at the pole of *L. capsici* AZ78 cells grown on Pea Agar Medium amended with 0.5% Agar (w/v; **A,C**) and these structures are absent in cells grown on Luria Bertani Agar amended with 0.5% Agar (w/v; **B,D**). A and B, magnification X-19000, black bar 500 nm. **(C,D)**, magnification X-64000, white bar 150 nm.

Finally, TEM analysis confirmed the presence of surface appendages on *L. capsici* AZ78 cells. The negative staining of *L. capsici* AZ78 cells revealed pilus-like structures localized at the poles of bacterial cells grown on PAM 0.5 (**Figures 6A,C**). In contrast, these structures were not present when *L. capsici* AZ78 was grown on LBA 0.5 (**Figures 6B,D**).

## Discussion

The efficacy of bacterial biocontrol agents is strictly associated with their ability to actively colonize the same ecological niches occupied by phytopathogenic microorganisms (Weller, 1988; Compant et al., 2005). To reach these niches, bacteria move in the environment thanks to external appendages, such as flagella and T4P (Mattick, 2002; Harshey, 2003; Kearns, 2010). Thus, determination of the mechanisms involved in the motility of bacterial biocontrol agents and understanding the factors that affect these mechanisms should be taken in consideration in the development of novel biopesticides. Unfortunately, little is known about the motility of *Lysobacter* members, a bacterial genus of increasing relevance in the development of biocontrol agents for important plant pathogens (Ji et al., 2008; Postma et al., 2010; Puopolo et al., 2014a,b). Recently, low control of *Rhizoctonia solani in vivo* by *Lysobacter* strains was associated with the poor ability of these strains to actively colonize the rhizosphere of different plants (Gómez Expósito et al., 2015). Understanding the factors and mechanisms involved in *Lysobacter* spp. motility is therefore crucial in order to improve their biocontrol efficacy.

Recently, we observed the dispersal of *L. capsici* AZ78 cells when grown on a medium containing PB used in tests aimed at evaluating *in vitro* inhibition of *P. infestans* (**Supplementary Figure S1**; Puopolo et al., 2014a). Based on these observations, we carried out greenhouse trials to discover whether PB could enhance the efficacy of *L. capsici* AZ78 against *P. viticola* by fostering cell movement on grapevine leaves. Interestingly, co-application of PB increased the quantity of *L. capsici* AZ78 cells residing on grapevine leaves and its efficacy against the phytopathogen significantly more than the bacterial strain applied alone. We carried out independent greenhouse trials to assess whether PB applied to grapevine leaves may lead to *L. capsici* AZ78 cell movement *in planta*. The results of the greenhouse experiments clearly showed that the quantity of PB remaining on grapevine leaves was sufficient to allow *L. capsici* AZ78 motility on agar surfaces.

Since these results could depend on the production of external appendages involved in the attachment of bacterial cells to the leaf and hyphae of phytopathogenic fungi and oomycetes (Van Doorn et al., 1994; Ojanen-Reuhs et al., 1997; Islam et al., 2005; Patel et al., 2011), we carried out further *in vitro* motility trials to better characterize the cell dispersal observed in *L. capsici* AZ78. We included other bacterial strains belonging to various *Lysobacter* species to have a more comprehensive analysis of cell motility in the *Lysobacter* genus.

The swimming and swarming tests carried out on SWM and SWR media confirmed that the type strains of *Lysobacter* spp. tested were unable to move on agar surfaces. Inability to move on these media containing 0.3 (SWM) and 0.5% (SWR) of agar was also observed in the case of type strains of the *L. arseniciresistens* and *L. spongiicola* species, although they have a single polar flagellum (Romanenko et al., 2008; Luo et al., 2012). The inability to move can be explained by the tendency of these bacterial strains to easily lose the flagella (Romanenko et al., 2008; Luo et al., 2012).

Inability to move on agar surfaces was also confirmed when most of the *Lysobacter* type strains were grown on PAM 0.3 and 0.5. In contrast, all the *L. capsici* strains moved on these two media, giving rise to macrocolonies characterized by the production of dendrites, which allowed rapid colonization of the medium surface after 20 h of incubation. Although the formation of dendrites is a typical trait of swarming motility in other bacterial species belonging to other genera (Harshey, 1994), to the best of our knowledge, this is the first report regarding dendrite formation in members of the *L. capsici* species.

In addition, we observed the formation of a biosurfactant ring surrounding the macrocolony in all *L. capsici* strains grown on PAM 0.5. Interestingly, the biosurfactant ring surrounding the macrocolony is another typical trait of swarming motility characterizing other bacterial strains belonging to other genera, as in the case of *B. subtilis* 3610 (Julkowska et al., 2004). Biosurfactants are involved in the swarming motility of many bacteria, such as *P. aeruginosa* (Köhler et al., 2000; Déziel et al., 2003), *B. subtilis* (Kearns and Losick, 2003) and *Serratia liquefaciens* (Lindum et al., 1998), where they help to overcome surface tension for efficient bacterial surface colonization (Matsuyama and Nakagawa, 1996; Harshey, 2003; Daniels et al., 2004). In contrast with other bacterial species, little attention has been paid to biosurfactant production in *Lysobacter* members. The only evidence of biosurfactant production was provided by Folman et al. (2004), who proved surfactant production in *L. enzymogenes* 3.1T8 cells grown in 1/2 and 1/10 Tryptone Soy Broth. However, biosurfactant compounds have not yet been identified and characterized in any *Lysobacter* member, and their characterisation merits additional studies.

Conventionally, swarming motility is described as flagella-related motility that allows the rapid colonization of semisolid surfaces (Harshey, 1994; Daniels et al., 2004). However, Köhler et al. (2000) and Overhage et al. (2007) reported the involvement of T4P in swarming motility of *P. aeruginosa* PAO1. A *fliC* mutant of *P. aeruginosa* PAO1 (PT690), unable to synthesize any flagellum, showed the ability to propagate on a semisolid medium while maintaining swarming motility (Köhler et al., 2000). T4P is also involved in twitching motility (Mattick, 2002) and this motility was recently reported in *L. enzymogenes* C3 and OH11 (Zhou et al., 2015). Interestingly, we observed that *L. enzymogenes* DSM 2043^T^ was moving through twitching on LBA 1, and a further increase in the colony area was recorded when this type strain was grown on PAM 1. These data thus confirm the twitching motility of members of the *L. enzymogenes* species and also show that PAM has some effect on the twitching motility of the *L. enzymogenes* type strain. Most of the other tested *Lysobacter* type strains did not move through twitching on either LBA 1 or PAM 1, with the sole exception of *L. capsici* strains. Indeed, a colony growth zone was observed on the bottom of plastic dishes when *L. capsici* strains were inoculated on PAM 1. Overall, the results of swimming, swarming and twitching assays indicated that *L. capsici* strains move on inert surfaces and this motility depended on the composition of the medium. This medium-dependent behavior was already observed in medium-dependent biofilm production by *L. capsici* AZ78 (Puopolo et al., 2014b).

Since the observed medium-dependent motility of *L. capsici* strains could be associated with the presence of both flagella and T4P, we mined the *L. capsici* AZ78 genome for genes involved in the production of these bacterial external appendages. We also assessed the involvement of these genes in the *L. capsici* AZ78 medium-dependent motility using qRT-PCR, since attempts to generate knock-out mutants have not been successful to date in the case of *L. capsici* strains (de Bruijn et al., 2015). The flagellar regulon in *L. capsici* AZ78 encompasses genes sharing a high amino acid sequence identity with flagellar proteins of *L. capsici* 55 (de Bruijn et al., 2015) and the related *Xc* and *Sm* species (Hayward et al., 2010). However, the *L. capsici* AZ78 genome lacked genes involved in regulation of the flagellar assembly pathway, such as the master operon *flhDC* (Liu and Matsumura, 1994), the anti-sigma factor *flgM* (Ohnishi et al., 1992) and a gene encoding the flagellin protein FliC. The lack of a functional regulon responsible for flagellum biogenesis was recently reported in other strains belonging to the *L. capsici*, *L. enzymogenes*, and *L. gummosus* species (de Bruijn et al., 2015). In spite of lack of functionality of the flagellum regulon, the *L. capsici* AZ78 genome has the protein export system of flagellar proteins (e.g., *flhA*, *flhB*, *fliH*, *fliI*, *fliP*, *fliQ*, and *fliR*). The presence of this export system, associated with the low expression level of putative *flhB*, *fliH*, *fliR*, and a non-functional flagellar biosynthesis pathway, led to the hypothesis that the flagellar system in *L. capsici* AZ78 evolved in a new function connected to protein export (Toft and Fares, 2008). This point may be involved in other aspects of their lifestyle, such as the establishment of pathogenic interaction with the microbial host (de Bruijn et al., 2015).

The T4P biogenesis system of *L. capsici* AZ78 is highly conserved in members of *Xc*, *Sm*, and other *Lysobacter* species. *L. capsici* AZ78 encompasses two *pilA* genes that were next to the putative *pilR-pilS* two-component system and followed by putative *pilB, pilC*, and *pilD* in a conserved gene cluster. The presence of two *pilA* genes has already been reported in *X. axonopodis* pv. *citri* strain 306 (*xac*3240 and *xac*3241; da Silva et al., 2002; Dunger et al., 2014). Moreover, another gene (AZ78_3612) encoding a pilin-like protein was identified in *L. capsici* AZ78, but it is probably not a *bona fide* T4P pilin, as previously reported for *X. axonopodis* pv. *citri* strain 306 (XAC3805) (Dunger et al., 2014).

In *P. aeruginosa* strains, the regulation of *pilA* transcription is controlled by RpoN (𝔖^54^) and the two-component system PilR–PilS (Ishimoto and Lory, 1989, 1992; Burrows, 2012). Interestingly, the growth of *L. capsici* AZ78 on PAM 0.5 was associated with the induction of both *rpoN* and *pilA* genes, as compared with LBA 0.5 and SWR 0.5, indicating a direct effect of the medium composition on T4P biogenesis. The movement of *L. capsici* AZ78 on PAM 0.5 was also associated with upregulation of a transcription factor (*clp*) involved in flagellar and T4P biogenesis of *Xc* and *L. enzymogenes*, respectively, (Lee et al., 2003; He et al., 2007; Wang et al., 2014).

Since the chemosensory system is responsible for regulating pilus assembly and retraction (Burrows, 2012; Leighton et al., 2015), the expression of genes belonging to the chemosensory Pil-Chp system (*pilI*, *pilG*, *pilJ*, and *chpA*) was assessed in *L. capsici* AZ78. The expression of *pilG* was upregulated on PAM 0.5, compared with the other two media tested. PilG is a CheY-like regulator that influences T4P biogenesis in *P. aeruginosa* (Fulcher et al., 2010) and *L. enzymogenes* C3 (Zhou et al., 2015). Similarly, the *pilJ* gene was induced in *L. capsici* AZ78 grown on PAM 0.5 and this gene encodes a MCP protein responsible for sensing environmental stimuli and inducing pilus extension (DeLange et al., 2007; Burrows, 2012). The flagellar chemotaxis-system plays an important role in swarming motility in response to environmental signals (Harshey, 2003; Daniels et al., 2004). For instance, swarming in *P. aeruginosa* PAO1 is induced by specific amino acids (glutamate and aspartate) and carbon sources (glucose and glycerol) (Köhler et al., 2000). Likewise, T4P-dependent “social gliding” is controlled by the chemotaxis-like system in *M. xanthus* and *Synechocystis* spp. (Shi and Zusman, 1995; Bhaya et al., 2001). Based on the expression profiles of *L. capsici* AZ78 genes involved in the chemotaxis-system, it is conceivable that peas release some compounds that are perceived by *L. capsici* strains, and then the expression of genes involved in T4P biogenesis favoring cell motility is triggered. This is also supported by the fact that the quantity of peas used in PAM affects *L. capsici* AZ78 motility and stimulates dendrite production. Similarly, *B. amyloliquefaciens* S499 can sense components of the plant cell wall (xylan and arabinogalactan) and this perception stimulates the production of the cyclic lipopeptide surfactin involved in biofilm formation and swarming motility (Debois et al., 2015).

Finally, the high expression level of genes responsible for the structure and regulation of T4P genes was associated with the presence of external appendages in *L. capsici* AZ78 grown on PAM 0.5 and visualized through TEM. In agreement with the gene expression levels, these structures were not visible on LBA 0.5-grown *L. capsici* AZ78 cells. The presence of external appendages (polar brush-like fimbriae) has already been reported in another biocontrol *Lysobacter* strain (*Lysobacter* sp. SB-K88) when grown on the roots of sugar beet seedlings (Islam et al., 2005), supporting the theory that the medium-dependent motility of *L. capsici* strains possibly relies on T4P.

## Conclusion

This work is a first step in deciphering motility mechanisms in *L. capsici* AZ78. The medium-dependent motility observed is associated with the release of a surfactant and with upregulation of the genes responsible for T4P biogenesis. This type of movement seems to be on the narrow borderline separating the swarming motility observed in *P. aeruginosa* (Köhler et al., 2000) and the “social gliding” of *M. xanthus* (Wu et al., 1997). From a practical point of view, the application of PB on grapevine plants increased efficacy against downy mildew, which is associated with an increase in leaf colonization by *L. capsici* AZ78. These results demonstrate that nutritional components could be used to improve the poor plant colonization observed in some *Lysobacter* strains (Gómez Expósito et al., 2015), and future studies aiming at identifying PAM piliation factors to be included in the formulation of *L. capsici* AZ78 may help to improve biocontrol efficacy under field conditions.

## Author Contributions

ST carried out all the experiments, analyzed the data and wrote and edited the manuscript. GP conceived the work, designed the experiments, carried out the greenhouse experiments, analyzed the data and wrote and edited the manuscript. RM carried out TEM analysis and wrote and edited the manuscript. MP, NL, and IP contributed to the conception of the work, designed the experiments and edited the manuscript. All the authors have read the manuscript and agreed to its content.

## Conflict of Interest Statement

The authors declare that the research was conducted in the absence of any commercial or financial relationships that could be construed as a potential conflict of interest.

## Footnotes

1 http://bioinfo.ut.ee
