# Supplementary material for: Pea Broth Enhances the Biocontrol Efficacy of Lysobacter capsici AZ78 by Triggering Cell Motility Associated with Biogenesis of Type IV Pilus
Source: Front Microbiol. 2016 Jul 26;7:1136. doi: 10.3389/fmicb.2016.01136 (PMC4960238; doi:10.3389/fmicb.2016.01136)
Supplement: Supplementary file 1 [file Table_1.PDF]

Table S1. Primers used in quantitative real-time polymerase chain reaction

Gene	Gene ID[1]	Primer Name	Primer Sequence (5'-3')	Amplicon Size (bp)
recA	AZ78_1089	recAF	GAGCCAGATCGACAAGCAAT	159
		recAR	GGACCGTAGATCTCGACCAC	
chpA	AZ78_4391	chpAF	GACCCGTCAGTACGAAAC	147
		chpAR	CAGTTCACTCGAAGCCTG	
clp	AZ78_4111	clpF	ACTCGGCCTATTCATCGAAA	108
		clpR	GTCAGCAGCAGGTCGTACAG	
rpoN	AZ78_1752	rpoNF	GAATGCCTCAACCTGCAACT	139
		rpoNR	TGTCGAGCTTGAGTTCGTTG	
fliA	AZ78_1611	fliAF	TGGAAAGCATCGTCGACTC	182
		fliAR	CTGGATGATCAGCCTCAGC	
flgE	AZ78_1616	flgEF	GTCGGATTACGAGGTGATGG	159
		flgER	TCGATCGAGGTGATCGTGTA	
flgI	AZ78_1627	flgIF	ATCAGTTCGAAGGCGTTACC	151
		flgIR	GTGAAATCCGGCTCGTTTAG	
flhB	AZ78_1633	flhBF	ACCTCGGTCTACGTGTTGCT	169
		flhBR	GCAATTGCTGTTTCTGCTTG	
fliH	AZ78_1621	fliHF	ATAACGCCAACGCACAACTC	163
		fliHR	CACAAGCGCTTCATCAGTTC	
fliR	AZ78_1633	fliRF	GAACTGAGCATCGGTTTCGT	200
		fliRR	AGGTCCAGGGTGAAGAACAG	
fimX	AZ78_4971	fimXF	AGTCGCGCGTCTACCACTA	121
		fimXR	ATGTAGCTGCGGTCGATCTT	
pilA	AZ78_4276	pilA1F	CAAGAACATGGAGTGCCTCA	123
		pilA1R	GAGGCGCTGAAGCTATAACC	
	AZ78_4277	pilA2F	CGTACCAAGAACGCTGAGTG	120
		pilA2R	GAACTGGTAGCCGGTCAGAG	
pilB	AZ78_4268	pilBF	GGTTCACCCACGAGGAGAT	103
		pilBR	ACCTGATAGATGCCGGTACG	
pilG	AZ78_4387	pilGF	ACGATTCCAAGACCATCCGT	129
		pilGR	CGAAAATGATCTGTGGCTGC	
pilI	AZ78_4389	pilIF	ACCTCAAGCAGTTCCTCG	107
		pilIR	CAGTTCGTCGATCAGCAC	
pilJ	AZ78_4390	pilJF	GAAAAGGTGTCGTCGGATC	104
		pilJR	ATCGTGTTCATCGTCTGGGT	
pilM	AZ78_2054	pilMF	CTACGGTCTGAGCTACGAGG	104
		pilMR	GGACCATCGCTTCCTTGAAC	
pilQ	AZ78_2049	pilQF	GAAGCTGGGCAGAACGAAAA	118
		pilQR	GCTCCTTCATCTGGGTCACT	
pilY1	AZ78_1457	pilYF	CAGTACAACAAGGCGATCCA	113
		pilYR	CGCATCTTCTCGGTCTTTTC	
pilZ	AZ78_5337	pilZF	TCGTTGACGATCAAGGACAA	126
		pilZR	GGTCAGCAGCAGGAACACTT	

[1] Gene codes of the *Lysobacter capsici* AZ78 genome (JAJA02000000; Puopolo et al., 2016)
