# Supplementary material for: Pea Broth Enhances the Biocontrol Efficacy of Lysobacter capsici AZ78 by Triggering Cell Motility Associated with Biogenesis of Type IV Pilus
Source: Front Microbiol. 2016 Jul 26;7:1136. doi: 10.3389/fmicb.2016.01136 (PMC4960238; doi:10.3389/fmicb.2016.01136)
Supplement: Supplementary file 2 [file Table_2.PDF]

Table S2. Motility on inert surface of *Bacillus amyloliquefaciens* S499 and *Pseudomonas chlororaphis* M71.

Bacterial strain	Motility Assay[1]	Medium	Colony area (mm^2)[2]
B. amyloliquefaciens S499	Swimming	SWM	6233.7 ± 64.3
		PAM 0.3	6172.3 ± 74.2
	Swarming	LBA 0.5	279.9 ± 28.9[c]
		SWR	461.0 ± 51.8[b]
		PAM 0.5	6266.8 ± 54.4[a]
	Twitching	LBA 1	218.4 ± 71.5
		PAM 1	519.7 ± 104.6*
P. chlororaphis M71	Swimming	SWM	1230.2 ± 154.8
		PAM 0.3	5525.5 ± 427.5*
	Swarming	LBA 0.5	48857.4 ± 542.5[a]
		SWR	4332.0 ± 378.6[a]
		PAM 0.5	5543.1 ± 327.2[a]
	Twitching	LBA 1	176.1 ± 15.6
		PAM 1	361.1 ± 76.9*

[1]The swimming, swarming and twitching motility of *B. amyloliquefaciens* S499 and *P. chlororaphis* M71 was evaluated on Swimming Agar (SWM), Pea Agar Medium 0.3 (PAM 0.3), Luria Bertani Agar 0.5 (LBA 0.5), Swarming agar (SWR), Pea Agar Medium 0.5 (PAM 0.5), Luria Bertani Agar 1 (LBA 1), Pea Agar Medium 1 (PAM 1).

[2] Mean colony area ± standard error values are calculated as the pool of nine replicates (Petri dishes) from three experiments. Value followed by asterisks indicate values that differ significantly according to Student's T test ($\alpha = 0.05$) in the pairwise comparison of SWM against PAM 0.3, and LBA 1 against PAM 1. Different letters indicate significant differences according to Tukey's test ($\alpha = 0.05$).
