# Supplementary material for: Pea Broth Enhances the Biocontrol Efficacy of Lysobacter capsici AZ78 by Triggering Cell Motility Associated with Biogenesis of Type IV Pilus
Source: Front Microbiol. 2016 Jul 26;7:1136. doi: 10.3389/fmicb.2016.01136 (PMC4960238; doi:10.3389/fmicb.2016.01136)
Supplement: Supplementary file 3 [file Table_3.PDF]

Table S3. Gene cluster of flagellum in *Lysobacter capsici* AZ78. Genetic organization of the gene cluster that includes putative genes encoding for the flagellar apparatus and hypothetical protein.

Gene	Function	Gene ID	Identity	E-value	Homologs/Orthologs	Gene ID
-	hypothetical protein	AZ78_1605			No putative conserved domains have been detected	
-	hypothetical protein	AZ78_1606			No putative conserved domains have been detected	
-	hypothetical protein	AZ78_1607	86%	0	hypothetical protein STRNTR1_1505 [*Stenotrophomonas maltophilia*]	KMU66257
-	putative enzyme; Integration, recombination (Phage or Prophage Related)	AZ78_1608	81%	0	hypothetical protein [*Stenotrophomonas maltophilia*]	WP_049443893
-	putative site specific recombinase	AZ78_1609	87%	0	integrase [*Stenotrophomonas maltophilia*]	WP_049397666
-	Single-stranded DNA-binding protein	AZ78_1610	96%	0	single-stranded DNA-binding protein [*Lysobacter arseniciresistens* ZS79]	KGM56886
			86%	0	single-stranded DNA-binding protein [*Stenotrophomonas maltophilia*]	KOO78064
			79%	0	MULTISPECIES: single-strand-binding protein [*Xanthomonas*]	WP_005989636
fliA	RNA polymerase sigma factor for flagellar operon	AZ78_1611	99%	3E-162	RNA polymerase sigma factor, sigma-70 family protein [*Lysobacter capsici* 55]	ALN84773
			48%	4E-54	hypothetical protein VO93_05635 [*Stenotrophomonas maltophilia*]	KOO80989
			40%	3.8E-02	RNA polymerase, sigma 70 (sigma D) factor [*Stenotrophomonas maltophilia*]	CRD57622
			39%	7E-40	RNA polymerase sigma factor, sigma-70 family [*Lysobacter enzymogenes* C3]	ALN59990
			37%	7E-33	RNA polymerase sigma-70 factor [*Xanthomonas campestris*]	KOB02991
fliP	Flagellar biosynthesis protein FliP	AZ78_1612	99%	6E-164	flagellar biosynthetic protein fliP	ALN84772

					[*Lysobacter capsici* 55]	
			72%	1E-114	flagellar biosynthesis protein fliP [*Stenotrophomonas maltophilia*]	KOO80988
			65%	8E-89	MULTISPECIES: flagellar biosynthetic protein FliP [*Xanthomonas*]	WP_016903031
			58%	6E-81	flagellar biosynthetic protein FliP [*Hahella chejuensis* KCTC 2396]	ABC30780
-	hypothetical protein	AZ78_1613			No putative conserved domains have been detected	
fliN	hypothetical protein	AZ78_1614	98%	3E-61	surface presentation of antigens family protein [*Lysobacter capsici* 55]	ALN84770
			70%	9E-31	hypothetical protein VO93_05620 [*Stenotrophomonas maltophilia*]	KOO80987
			50%	3E-16	hypothetical protein AE921_04220 [*Xanthomonas campestris*]	AKU51567
			45%	4E-15	flagellar motor switch protein FliN [*Pseudomonas fluorescens*]	KIQ61287
fliM	hypothetical protein	AZ78_1615	99%	5E-174	surface presentation of antigens family protein [*Lysobacter capsici* 55]	ALN84769
			30%	1E-27	hypothetical protein VO93_05615 [*Stenotrophomonas maltophilia*]	KOO78089
flgE	Flagellar hook protein FlgE	AZ78_1616	99%	0	flagellar hook-basal body protein [*Lysobacter capsici* 55]	ALN84768
			60%	2E-152	flagellar hook-basal body protein [*Stenotrophomonas maltophilia*]	KOO78088
			55%	3E-130	flagellar hook-basal body protein [*Xanthomonas campestris*]	KOB02987
			49%	6E-106	flagellar hook protein FlgE [*Pseudomonas fluorescens*]	WP_024617155
flgD	Flagellar basal-body rod modification protein FlgD	AZ78_1617	100%	7E-92	flagellar hook capping N-terminal region family protein [*Lysobacter capsici* 55]	ALN84767
			63%	2E-51	flagellar hook capping protein [*Stenotrophomonas maltophilia*]	KOO78087
			58%	1E-42	flagellar hook capping protein	KOB02986

					[*Xanthomonas campestris*]	
			41%	7E-25	Flagellar hook capping protein [*Hahella chejuensis* KCTC 2396]	ABC30785
-	hypothetical protein	AZ78_1618			No putative conserved domains have been detected	
-	hypothetical protein	AZ78_1619			No putative conserved domains have been detected	
fliI	Flagellum-specific ATP synthase FliI	AZ78_1620	100%	0	ATPase FliI/YscN family protein [*Lysobacter capsici* 55]	ALN84764
			67%	0	ATP synthase [*Stenotrophomonas maltophilia*]	KOO78084
			59%	4E-168	ATP synthase [*Xanthomonas campestris*]	KOB02983
			55%	3E-152	flagellum-specific ATP synthase [*Hahella chejuensis* KCTC 2396]	ABC30788
fliH	Flagellar assembly protein FliH	AZ78_1621	97%	1E-148	flagellar assembly FliH family protein [*Lysobacter capsici* 55]	ALN84763
			36%	2E-15	hypothetical protein VO93_05585 [*Stenotrophomonas maltophilia*]	KOO78083
			31%	2E-14	hypothetical protein AE921_04185 [*Xanthomonas campestris*]	KOB02982
			31%	4E-07	flagellar assembly protein FliH [*Pseudomonas* sp. GM48]	WP_007988851
-	hypotetical protein	AZ78_1622			No putative conserved domains have been detected	
fliF	Flagellar M-ring protein FliF	AZ78_1623	99%	0	flagellar M-ring protein FliF [*Lysobacter capsici* 55]	ALN84761
			61%	0	hypothetical protein VO93_05575 [*Stenotrophomonas maltophilia*]	KOO80986
			44%	4E-103	flagellar M-ring protein FliF [*Xanthomonas campestris*]	KOB03079
			34%	2E-68	flagellar M-ring protein FliF [*Hahella chejuensis* KCTC 2396]	ABC30791
fliE	Flagellar hook-basal body complex	AZ78_1624	100%	2E-68	flagellar hook-basal body complex protein	ALN84760

	protein FliE				FliE [*Lysobacter capsici* 55]	
			50%	3E-25	hypothetical protein VO93_05570 [*Stenotrophomonas maltophilia*]	KOO78081
			49%	9E-24	MULTISPECIES: flagellar hook-basal body protein FliE [*Xanthomonas*]	WP_016849718
			42%	1E-14	flagellar hook-basal body complex protein FliE [*Hahella chejuensis* KCTC 2396]	ABC30792
flgC	Flagellar basal-body rod protein FlgC	AZ78_1625	100%	2E-86	Flagellar basal body rod FlgEFG C-terminal family protein [*Lysobacter capsici* 55]	ALN84759
			62%	1E-46	hypothetical protein VO93_05565 [*Stenotrophomonas maltophilia*]	KOO78080
			58%	1E-38	MULTISPECIES: hypothetical protein [*Xanthomonas*]	WP_003488392
			43%	1E-27	MULTISPECIES: flagellar basal-body rod protein FlgC [*Pseudomonas*]	WP_042727962
flgB	hypotetical protein	AZ78_1626	98%	8E-78	hypothetical protein LC55x_1476 [*Lysobacter capsici* 55]	ALN84758
			47%	9E-22	MULTISPECIES: hypothetical protein [*Xanthomonas*]	WP_039814212
			42%	7E-15	hypothetical protein VO93_05560 [*Stenotrophomonas maltophilia*]	KOO78079
			25%	9.4E-02	Flagellar basal body protein [*Hahella chejuensis* KCTC 2396]	ABC30794
flgI	Flagellar P-ring protein FlgI	AZ78_1627	100%	0	flagellar P-ring protein FlgI [*Lysobacter capsici* 55]	ALN84757
			75%	0	flagellar P-ring protein FlgI [*Stenotrophomonas maltophilia*]	KOO80985
			70%	3E-170	flagellar P-ring protein FlgI [*Xanthomonas campestris*]	KOB02978
			55%	8E-125	Flagellar P-ring protein 1; AltName: Basal body P-ring protein [*Hahella chejuensis* KCTC 2396]	Q2SEX9
flgH	Flagellar L-ring protein FlgH	AZ78_1628	99%	4E-143	flagellar L-ring protein	ALN84756

					[*Lysobacter capsici* 55]	
			71%	9E-82	flagellar L-ring protein FlgH [*Stenotrophomonas maltophilia*]	KOO78078
			55%	6E-56	flagellar L-ring protein FlgH [*Xanthomonas campestris*]	KOB02977
			37%	7E-26	flagellar basal body L-ring protein [*Hahella chejuensis*]	WP_011397863
flgA	Flagellar basal-body P-ring formation protein FlgA	AZ78_1629	98%	9E-156	Flagellar basal body P-ring formation proteinFlgA [*Lysobacter capsici* 55]	ALN84755
			43%	2E-43	hypothetical protein ARC02_03325 [*Stenotrophomonas maltophilia*]	KRG61249
			36%	2E-19	flagellar biosynthesis protein FlgA [*Xanthomonas campestris*]	WP_053054111
flgG	Flagellar basal-body rod protein FlgG	AZ78_1630	99%	0	flagellar basal-body rod protein FlgG [*Lysobacter capsici* 55]	ALN84754
			62%	1E-103	flagellar hook-basal body protein [*Stenotrophomonas maltophilia*]	KOO78077
			56%	1E-89	flagellar hook-basal body protein [*Xanthomonas campestris*]	KOB02975
flgF	Flagellar basal-body rod protein FlgF	AZ78_1631	99%	7E-156	Flagellar hook-basal body family protein [*Lysobacter capsici* 55]	ALN84753
			58%	4E-81	hypothetical protein ARC02_03335 [*Stenotrophomonas maltophilia*]	KRG61251
			46%	4E-59	flagellar hook-basal body protein [*Xanthomonas campestris*]	KOB02974
			33%	6E-27	flagellar basal body rod protein FlgG [*Pseudomonas fluorescens*]	WP_024617143
flhA	Flagellar biosynthesis protein FlhA	AZ78_1632	100%	0	flagellar biosynthesis protein FlhA [*Lysobacter capsici* 55]	ALN84752
			76%	0	flagellar biosynthesis protein FlhA [*Stenotrophomonas maltophilia*]	KOO78075
			67%	0	flagellar biosynthesis protein FlhA [*Xanthomonas campestris*]	KOB16242
			52%	0	Flagellar biosynthesis pathway,	ABC30800

					component FlhA [*Hahella chejuensis* KCTC 2396]	
flhB	Flagellar biosynthesis protein FlhB	AZ78_1633	99%	0	flhB HrpN YscU SpasS family protein [*Lysobacter capsici* 55]	ALN84751
			66%	5E-150	flagellar biosynthesis protein FlhB [*Stenotrophomonas maltophilia*]	KRG61253
			53%	8E-114	flagellar biosynthesis protein FlhB [*Xanthomonas arboricola*]	WP_039814221
			44%	1E-82	flagellar biosynthesis protein [*Pseudomonas fluorescens*]	KWU52851
fliR	Flagellar biosynthesis protein FliR	AZ78_1634	99%	3E-173	bacterial export, 1 family protein [*Lysobacter capsici* 55]	ALN84750
			63%	1E-101	hypothetical protein ARC02_03350 [*Stenotrophomonas maltophilia*]	KRG61254
			57%	7E-73	UDP kinase [*Xanthomonas campestris*]	KOB02972
			40%	8E-23	flagellar biosynthesis protein [*Pseudomonas fluorescens*]	KWU52850
			33%	2E-23	Flagellar biosynthesis pathway, component FliR [*Hahella chejuensis* KCTC 2396]	ABC30802
fliQ	Flagellar biosynthesis protein FliQ	AZ78_1635	100%	1E-50	Bacterial export, 3 family protein [*Lysobacter capsici* 55]	ALN84749
			79%	2E-40	flagellar biosynthetic protein FliQ [*Stenotrophomonas maltophilia*]	KRG61255
			69%	1E-27	export protein FliQ [*Xanthomonas campestris*]	KOB03078
			57%	6E-02	flagellar biosynthetic protein FliQ [*Hahella chejuensis* KCTC 2396]	ABC30803
			55%	2E-17	export protein FliQ [*Pseudomonas fluorescens*]	KIQ61315
-	TPR domain protein, putative component of TonB system	AZ78_1636	99%	0	TPR repeat family protein [*Lysobacter capsici* 55]	ALN84748
			55%	4E-118	hypothetical protein ARC02_03360 [*Stenotrophomonas maltophilia*]	KRG61256

			48%	8E-45	hypothetical protein AE923_03890, partial [*Xanthomonas campestris*]	KOB11384
-	hypothetical protein	AZ78_1637			No putative conserved domains have been detected	
-	hypothetical protein	AZ78_1638	60%	2E-53	hypothetical protein ARC02_03370 [*Stenotrophomonas maltophilia*]	KRG61258
			45%	6E-30	hypothetical protein AE921_13040 [*Xanthomonas campestris*]	KOA99158
