# Supplementary material for: Pea Broth Enhances the Biocontrol Efficacy of Lysobacter capsici AZ78 by Triggering Cell Motility Associated with Biogenesis of Type IV Pilus
Source: Front Microbiol. 2016 Jul 26;7:1136. doi: 10.3389/fmicb.2016.01136 (PMC4960238; doi:10.3389/fmicb.2016.01136)
Supplement: Supplementary file 4 [file Table_4.PDF]

Table S4. Gene clusters of Type 4 pilus in *Lysobacter capsici* AZ78. Genetic organization of the gene clusters that includes putative genes encoding for T4P and hypothetical protein.

Gene		Function	Gene ID	Identity	E-value	Homologs/Orthologs	Gene ID
	pilE	Type IV pilus biogenesis protein PilE	AZ78_1456	100%	3E-102	prepilin-type N-terminal cleavage/methylation domain protein [*Lysobacter capsici* 55]	ALN84946
				63%	6E-38	putative type IV pilin [*Lysobacter antibioticus* 76]	ALN79896
				55%	3E-37	type IV pilin [*Stenotrophomonas maltophilia*]	KRG55821
				52%	9E-35	Pilus assembly protein PilE [*Xanthomonas campestris*]	WP_039407505
	pilY1	Type IV fimbrial biogenesis protein PilY1	AZ78_1457	96%	0	neisseria PilC beta-propeller domain protein [*Lysobacter capsici* 55]	ALN84945
				73%	0	neisseria PilC beta-propeller domain protein [*Lysobacter gummosus* 3.2.11]	ALN92492
				44%	0	putative PilY1 protein [*Xanthomonas campestris pv. viticola*]	CDN19294
				43%	0	PilY1 protein [*Stenotrophomonas maltophilia* AU12-09]	EMI50639
**C**				43%	0	Tfp pilus assembly protein, tip-associated adhesin PilY1 [*Pseudomonas aeruginosa*]	CRQ55241
**L**	*pilX*	Type IV fimbrial biogenesis protein PilX	AZ78_1458	99%	1E-105	pilX N-terminal family protein [*Lysobacter capsici* 55]	ALN84944
**U**				81%	8E-78	pilX N-terminal family protein [*Lysobacter antibioticus* 76]	ALN79894
**S**				49%	1E-22	protein PilX [*Stenotrophomonas maltophilia*]	ALA90019
**T**				43%	3E-23	PilX protein [*Xanthomonas campestris* pv. *campestris* str. ATCC 33913]	NP_637842

**E**				42%	2E-18	Tfp pilus assembly protein PilX [*Pseudomonas aeruginosa*]	CRQ55258
**R**	*pilW*	Type IV fimbrial biogenesis protein PilW	AZ78_1459	98%	0	putative pilW [*Lysobacter capsici* 55]	ALN84943
				69%	0	putative pilW [*Lysobacter gummosus* 3.2.11]	ALN92494
**1**				44%	4E-89	pilus assembly protein PilW [*Xanthomonas campestris*]	KOA99459
				44%	1E-82	pilus assembly protein PilW [*Stenotrophomonas maltophilia*]	KRG52456
				43%	1E-81	PilW-related protein [[*Pseudomonas*] *geniculata*]	WP_010486095
	pilV	Type IV fimbrial biogenesis protein PilV	AZ78_1460	91%	1E-83	type IV pilus modification protein PilV [*Lysobacter capsici* 55]	ALN84942
				70%	3E-57	type IV pilus modification protein PilV [*Lysobacter gummosus* 3.2.11]	ALN92495
				49%	3E-31	pilus assembly protein PilV [*Stenotrophomonas maltophilia*]	WP_049466992
				45%	4E-32	pilus assembly protein PilV [*Xanthomonas campestris pv. campestris*]	AKS15873
				44%	5E-26	type IV pilus modification protein PilV [*Pseudomonas aeruginosa*]	CRQ55296
	fimU	Type IV fimbrial biogenesis protein FimT	AZ78_1461	98%	3E-113	type II transport GspH family protein [*Lysobacter capsici* 55]	ALN84941
				59%	5E-56	prepilin-type N-terminal cleavage/methylation domain protein [*Lysobacter antibioticus* ATCC 29479]	ALN65104
				43%	2E-35	pre-pilin like leader sequence [*Stenotrophomonas maltophilia*]	W_049466990
				40%	2E-28	putative type IV pilus assembly protein FimT [*Stenotrophomonas maltophilia*]	CRD47827
				40%	3E-28	type IV pilus assembly protein FimT [*Xanthomonas axonopodis* pv. *malvacearum* str. GSPB2388]	EKQ62306

				39%	9E-32	pre-pilin like leader sequence [*Xanthomonas campestris*]	KOR71165
				33%	1E-24	putative type 4 fimbrial biogenesis protein FimU [*Stenotrophomonas maltophilia*]	AIU94607
				33%	1E-24	putative FimU protein (type 4 fimbrial biogenesis protein FimU) [*Stenotrophomonas maltophilia* K279a]	CAQ45152
	fimT	hypothetical protein	AZ78_1462	99%	5E-124	type II transport GspH family protein [*Lysobacter capsici* 55]	ALN84940
				32%	1E-10	type IV pilus assembly protein FimT [*Xanthomonas translucens* pv. *phlei*]	CTP83791
				32%	2E-09	pre-pilin like leader sequence [*Stenotrophomonas maltophilia*]	KRG41152
				31%	3E-14	putative type IV pilus assembly protein FimT [*Pseudoxanthomonas suwonensis* 11-1]	ADV26706
	pilE	pilus biogenesis protein PilE	AZ78_1503	100%	2E-55	putative prepilin like protein [*Lysobacter capsici* 55]	ALN84874
				49%	2E-19	pilus assembly protein PilE [*Stenotrophomonas maltophilia*]	KRG41270
				43%	6E-10	pilus assembly protein PilE [*Xanthomonas campestris*]	KIQ24652
				40%	1E-06	type IV pilus biogenesis protein [*Pseudomonas syringae* BRIP39023]	ELQ08607
	pilY1	Type IV fimbrial biogenesis protein PilY1	AZ78_1504	98%	0	neisseria PilC beta-propeller domain protein [*Lysobacter capsici* 55]	ALN84873
				46%	0	type IV pilus biogenesis protein PilY [*Xanthomonas translucens* pv. *poae*]	CTP84286
**C**				46%	0	PilY1 gene product [*Xylella fastidiosa* 9a5c]	AAF82845
**L**				44%	0	putative Tfp pilus assembly protein [*Xanthomonas campestris* pv. *campestris*]	CAP51021
**U**	*pilX*	Type IV fimbrial biogenesis protein PilX	AZ78_1505	99%	2E-124	PilX N-terminal family protein [*Lysobacter capsici* 55]	ALN84872

				47%	3E-34	PilX protein [*Xanthomonas arboricola*]	WP_047126760
**S**				48%	3E-35	conserved hypothetical protein [*Xanthomonas campestris* pv. *campestris*]	CAP51020
**T**				49%	2E-29	PilX protein [*Xylella fastidiosa* PLS229]	EWS79272
**E**	*pilW*	Type IV fimbrial biogenesis protein PilW	AZ78_1506	99%	0	hypotetical protein LC55x_1580 [*Lysobacter capsici* 55]	ALN84871
**R**				39%	6E-61	Tfp pilus assembly protein PilW [*Xanthomonas translucens* pv. *graminis* ART-Xtg29]	EKU25035
**2**				36%	2E-53	pilus assembly protein PilW [*Xylella fastidiosa*]	KXB11308
	pilV	Type IV fimbrial biogenesis protein PilV	AZ78_1507	100%	3E-76	type IV pilus modification protein PilV [*Lysobacter capsici* 55]	ALN84870
				45%	2E-19	type IV pilus modification protein PilV [*Xylella fastidiosa*]	WP_004085076
				44%	4E-13	prepilin [*Xanthomonas campestris* pv. *musacearum* NCPPB 4380]	KFA10075
				42%	4E-12	type IV pilus modification protein PilV [*Stenotrophomonas daejeonensis*]	WP_057639476
	fimU	type 4 fimbrial biogenesis protein FimU	AZ78_1508	99%	4E-109	type II transport GspH family protein [*Lysobacter capsici* 55]	ALN84869
				41%	5E-20	prepilin [*Xylella fastidiosa*]	WP_038269594
				40%	5E-21	prepilin [*Xanthomonas campestris* pv. *arecae*]	KGP59035
				36%	1E-15	type 4 fimbrial biogenesis protein FimU [*Stenotrophomonas acidaminiphila*]	ALJ27819
	-	hypothetical protein	AZ78_1509			No putative conserved domains have been detected	
	fimT	type 4 fimbrial biogenesis protein FimU	AZ78_1510	98%	1E-111	type II transport GspH family protein [*Lysobacter capsici* 55]	ALN84867
				48%	9E-39	type IV fimbrial biogenesis protein [*Lysobacter enzymogenes* C3]	ALN58972
				37%	5E-19	pilus assembly protein FimT [*Xanthomonas sacchari*]	WP_010342099

				35%	3E-21	pilus assembly protein [*Pseudoxanthomonas* sp. J35]	WP_028918125
				34%	2E-16	MULTISPECIES: hypothetical protein [*Stenotrophomonas*]	WP_055769968
	pilU	Twitching motility protein PilT	AZ78_1780	93%	0	twitching motility protein PilT [*Stenotrophomonas maltophilia*]	WP_049426723
**C**				92%	0	twitching motility protein [*Xanthomonas campestris* pv. *campestris* str. ATCC 33913]	NP_638102
**.**	*pilT*	Twitching motility protein PilT	AZ78_1781	94%	0	twitching motility protein PilT [*Stenotrophomonas maltophilia*]	WP_019185385
**3**				94%	0	twitching mobility protein [*Pseudomonas aeruginosa*]	CRQ51480
				94%	0	twitching motility protein PilT [*Xanthomonas campestris* pv. *campestris*]	AKS19670
	pilQ	Type IV pilus biogenesis protein PilQ	AZ78_2049	97%	0	type IV pilus secretin PilQ family protein [*Lysobacter capsici* 55]	ALN84333
				87%	0	type IV pilus secretin PilQ family protein [*Lysobacter antibioticus* ATCC 29479]	ALN65004
				83%	0	Type IV assembly protein [*Lysobacter enzymogenes* C3]	ALN56294
				63%	0	fimbrial assembly protein [*Xanthomonas campestris* pv. *campestris* str. ATCC 33913]	NP_638574
				62%	0	Type IV pilus biogenesis protein PilQ [*Stenotrophomonas maltophilia* D457]	CCH13940
**C**	-	hypothetical protein	AZ78_2050			No putative conserved domains have been detected	
**L**	*pilP*	Type IV pilus biogenesis protein PilP	AZ78_2051	98%	5E-127	pilus assembly, PilP family protein [*Lysobacter capsici* 55]	ALN84332
**U**				89%	2E-102	type IV assembly protein [*Lysobacter enzymogenes* C3]	ALN56293
**S**				86%	5E-111	pilus assembly, PilP family protein [*Lysobacter gummosus* 3.2.11]	ALN89954

**T**				73%	1E-84	fimbrial protein [*Stenotrophomonas maltophilia*]	WP_046274019
**E**				72%	5E-84	fimbrial protein [*Xanthomonas campestris*]	AKC79290
**R**	*pilO*	Type IV pilus biogenesis protein PilO	AZ78_2052	95%	9E-161	pilO protein [*Lysobacter capsici* 55]	ALN84331
				90%	8E-132	pilO protein [*Lysobacter antibioticus* ATCC 29479]	ALN65006
**4**				87%	2E-127	type IV assembly protein [*Lysobacter enzymogenes* C3]	ALN56292
				86%	4E-127	pilO protein [*Lysobacter gummosus* 3.2.11]	ALN89953
				69%	2E-104	fimbrial protein [*Stenotrophomonas maltophilia*]	WP_053518455
				75%	5E-112	Fimbrial assembly membrane protein [*Xanthomonas campestris* pv. *campestris*]	CAP50304
	pilN	Type IV pilus biogenesis protein PilN	AZ78_2053	99%	0	fimbrial assembly family protein [*Lysobacter capsici* 55]	ALN84330
				92%	1E-116	fimbrial assembly family protein [*Lysobacter antibioticus* 76]	ALN82271
				90%	4E-104	Type IV pilus assembly protein [*Lysobacter enzymogenes* C3]	ALN56291
				65%	1E-74	fimbrial protein [*Xanthomonas campestris* pv. *campestris*]	AKS19290
				61%	8E-73	fimbrial protein, partial [*Stenotrophomonas maltophilia*]	WP_019338751
	pilM	Type IV pilus biogenesis protein PilM	AZ78_2054	100%	0	type IV pilus assembly PilM family protein [*Lysobacter capsici* 55]	ALN84329
				98%	0	type IV pilus assembly PilM family protein [*Lysobacter antibioticus* ATCC 29479]	ALN65008
				97%	0	type IV pilus assembly protein [*Lysobacter enzymogenes* C3]	ALN56290
				82%	0	pilus assembly protein PilM	WP_042614422

				80%	0	[*Stenotrophomonas maltophilia*] pilus assembly protein PilM [*Xanthomonas campestris*]	AKC79293
**CLUSTER 5**	*pilD*	Leader peptidase (Prepilin peptidase)	AZ78_4266	90%	0	type IV prepilin-like proteins leader peptide-processing enzyme [*Lysobacter enzymogenes* C3]	ALN59460
				69%	7E-139	leader peptidase (prepilin peptidase) / N-methyltransferase [*Stenotrophomonas maltophilia* RA8]	CCP18068
				67%	1E-135	type IV pre-pilin leader peptidase [*Xanthomonas campestris* pv. *campestris* str. ATCC 33913]	NP_638448
	pilC	Type IV fimbrial assembly protein PilC	AZ78_4267	90%	0	type IV pilus assembly protein [*Lysobacter enzymogenes* C3]	ALN59459
				74%	0	pilC, fimbrial assembly protein [*Xanthomonas campestris* pv. *campestris*]	CAP50436
				73%	0	type IV fimbrial assembly protein PilC [*Stenotrophomonas maltophilia* EPM1]	EMF59361
	pilB	Type IV fimbrial assembly, ATPase PilB	AZ78_4268	98%	0	type IV-A pilus assembly, ATPase PilB [*Lysobacter capsici* 55]	ALN87507
				82%	0	type IV fimbrial assembly, ATPase PilB [*Lysobacter dokdonensis* DS-58]	KGQ18858
				76%	0	type 4 fimbrial assembly protein pilB [*Stenotrophomonas maltophilia*]	CRD61026
				75%	0	pilus biogenesis protein [*Xanthomonas campestris* pv. *campestris* str. ATCC 33913]	NP_638444
				74%	0	type 4 fimbrial assembly protein pilB [*Xanthomonas translucens*]	WP_009580420
	pilA.1	Type IV pilin PilA	AZ78_4276	59%	4E-30	prepilin-type N-terminal cleavage/ methylation domain protein [*Lysobacter capsici* 55]	ALN87506
				42%	5E-15	pilin [*Xanthomonas campestris*]	KOB01592
				31%	5E-04	fimbrillin	AAM38084

					[*Xanthomonas axonopodis* pv. *citri* str. 306]	
pilA.2	Type IV pilin PilA	AZ78_4277	73%	9E-07	Fimbrillin [*Xanthomonas axonopodis* pv. *citri* str. 306]	AAM38085
			68%	1E-43	Prepilin-type N-terminal cleavage/ methylation domain protein [*Lysobacter capsici* 55]	ALN87506
			56%	2E-27	pilin [*Xanthomonas campestris*]	KOB01592
			31%	5E-04	fimbrillin [*Xanthomonas axonopodis* pv. *citri* str. 306]	AAM38084
pilR	Type IV fimbriae expression regulatory protein PilR	AZ78_4278	100%	0	bacterial regulatory, Fis family protein [*Lysobacter capsici* 55]	ALN87505
			73%	0	PilR [*Xanthomonas campestris* pv. *campestris*]	AAP43028
			73%	0	two-component system regulatory protein [*Xanthomonas campestris* pv. *campestris* str. ATCC 33913]	NP_638443
			72%	0	two-component system response regulator [*Stenotrophomonas maltophilia*]	WP_049405671
pilS	Two-component sensor PilS	AZ78_4279	99%	0	his Kinase A domain protein [*Lysobacter capsici* 55]	ALN87504
			58%	0	two-component system sensor protein [*Xanthomonas campestris* pv. *campestris* str. ATCC 33913]	NP_638442
			58%	0	PilS [*Xanthomonas campestris* pv. *campestris*]	AAP43027
			58%	0	two-component system sensor histidine kinase [*Stenotrophomonas maltophilia*]	WP_017356171
comL	putative component of the lipoprotein assembly complex	AZ78_4284	100%	0	competence protein [*Lysobacter capsici* 55]	ALN87499
			88%	0	competence protein	ALN62868

						[*Lysobacter antibioticus* ATCC 29479]	
				71%	3E-139	competence protein [*Stenotrophomonas maltophilia*]	KWV53255
				67%	1E-138	competence protein [*Xanthomonas campestris*]	KOR70810
**CLUSTER 6**	*pilG*	twitching motility protein PilG	AZ78_4387	100%	1E-79	response regulator [*Lysobacter capsici* 55]	ALN87396
				98%	6E-78	type IV pilus assembly protein [*Lysobacter enzymogenes* C3]	ALN59304
				98%	1E-88	pilus assembly protein PilG [*Lysobacter enzymogenes*]	WP_057948678
				94%	1E-79	pilus assembly protein PilG [*Xanthomonas campestris*]	KOB02299
				92%	4E-78	pilus assembly protein PilG [*Stenotrophomonas maltophilia*]	KIP84116
				86%	7E-70	pilus assembly protein PilG [*Pseudomonas aeruginosa*]	KSF29090
	pilH	twitching motility protein PilH	AZ78_4388	100%	2E-81	response regulator [*Lysobacter capsici* 55]	ALN87395
				71%	6E-55	twitching motility protein PilH [*Stenotrophomonas maltophilia* D457]	CCH13759
				70%	6E-54	PilH protein [*Xanthomonas campestris* pv. *campestris* str. ATCC 33913]	NP_638272
				55%	1E-45	protein PilH [*Pseudomonas aeruginosa*]	WP_034007405
	pilI	type IV pili signal transduction protein PilI	AZ78_4389	100%	1E-127	cheW-like domain protein [*Lysobacter capsici* 55]	ALN87394
				65%	4E-68	chemotaxis protein CheW [*Stenotrophomonas maltophilia*]	WP_049400398
				62%	1E-66	chemotaxis protein CheW [*Xanthomonas campestris* pv. *campestris*]	WP_014508568
	pilJ	type IV pilus biogenesis protein PilJ	AZ78_4390	100%	0	methyl-accepting chemotaxis (MCP) signaling domain protein [*Lysobacter capsici* 55]	ALN87393
				67%	0	putative pilus biogenesis protein	CAQ47087

					PilJ/methyl accepting chemotaxis protein [*Stenotrophomonas maltophilia* K279a]	
			67%	0	pilus biogenesis protein [*Xanthomonas campestris* pv. *campestris* str. ATCC 33913]	NP_638270
chpA	Signal transduction histidine kinase CheA (EC 2.7.3.-)	AZ78_4391	92%	0	transcriptional regulator [*Lysobacter capsici* 55]	ALN87392
			69%	0	transcriptional regulator [*Stenotrophomonas maltophilia*]	WP_019185135
			51%	0	MULTISPECIES: sensor histidine kinase [*Xanthomonas*]	WP_022558749
			44%	0	PilL [*Xanthomonas campestris* pv. *campestris*]	ACF16411
chpB	glutamate methyltransferase	AZ78_4392	53%	2E-39	chemotaxis protein [*Stenotrophomonas maltophilia*]	WP_043399184
			48%	4E-37	chemotaxis protein [*Xanthomonas campestris*]	WP_039412175
chpC	Chemotaxis signal transduction protein	AZ78_4393	55%	7E-42	putative chemotaxis CheW protein [*Stenotrophomonas maltophilia* K279a]	CAQ47084
			54%	1E-42	Chemotaxis-related protein [*Xanthomonas campestris* pv. *campestris*]	CAP50583
fimV	Tfp pilus assembly protein FimV	AZ78_0130	97	0	fimV N-terminal domain [*Lysobacter capsici* 55]	ALN86296
			71	0	fimV protein [*Lysobacter antibioticus* ATCC 29479]	ALN61076
			43	2E-60	fimV protein [*Stenotrophomonas maltophilia*]	WP_019336664
			43	4E-59	FimV protein [*Xanthomonas campestris* pv. *raphani* 756C]	AEL07747
pilF	Type IV pilus biogenesis protein PilF	AZ78_0791	100	0	type IV pilus biogenesis/stability protein [*Lysobacter capsici* 55]	ALN85602
			50	2E-53	fimbrial protein, partial [*Xanthomonas*	KFA34954

					campestris pv. *musacearum* NCPPB 4384]	
			49	9E-69	type IV pilus biogenesis protein PilF [*Lysobacter* sp. A03]	KIQ96141
rpoN	RNA polymerase sigma-54 factor RpoN	AZ78_1752	100	0	RNA polymerase sigma-54 factor [*Lysobacter capsici* 55]	ALN84614
			89	0	RNA polymerase sigma-54 factor [*Lysobacter gummosus* 3.2.11]	ALN90213
			88	0	RNA polymerase sigma-54 factor [*Lysobacter antibioticus* ATCC 29479]	ALN61594
			67	0	RNA polymerase sigma-54 factor [*Xanthomonas campestris* pv. *viticola*]	CDN18987
			65	0	RNA polymerase sigma54 factor [*Stenotrophomonas maltophilia*]	KOQ68432
pilA	Type IV pilin PilA	AZ78_3612	97	0	Pilin family protein [*Lysobacter capsici* 55]	ALN88174
			39	1E-28	xac3805 pilin like protein - not a bona fide pilin [*Xanthomonas axonopodis* pv. *citri* str. 306]	AAM38647
			37	2E-37	fimbrial protein [*Stenotrophomonas maltophilia*]	WP_017355384
			37	3E-40	hypothetical protein [*Xanthomonas campestris*]	WP_040941922
clp	Cyclic AMP receptor protein	AZ78_4111	99	1E-146	Clp regulator [*Lysobacter enzymogenes* C3]	AAP83141
			99	2E-126	CRP transcriptional dual regulator [*Lysobacter gummosus* 3.2.11]	ALN93145
			99	2E-126	CRP transcriptional dual regulator [Lysobacter capsici 55]	ALN87652
			84	2E-121	cAMP-regulatory protein [*Xanthomonas campestris* pv. *campestris*	NP_635866

					str. ATCC 33913]	
			84	7E-121	transcriptional regulator [*Stenotrophomonas maltophilia*]	WP_017354543
			83	7E-120	cAMP-regulatory protein [Stenotrophomonas maltophilia R551-3]	WP_010482649
			45	2E-71	vfr cAMP-regulatory protein [*Pseudomonas aeruginosa* PAO1]	NP_249343.1
fimX	two-component system response regulator	AZ78_4971	100	0	diguanylate cyclase domain protein [*Lysobacter capsici* 55]	ALN86593
			55	0	putative transmembrane protein [*Stenotrophomonas maltophilia* K279a]	CAQ45526
			55	0	Putative sensor protein [*Xanthomonas campestris* pv. *campestris*]	CAP51240
			54	0	diguanylate cyclase/phosphodiesterase [*Xanthomonas citri* subsp. *citri* A306]	AJD68992
pilZ	Type IV pilus biogenesis protein PilZ	AZ78_5337	99	5E-70	type IV fimbriae assembly protein [*Lysobacter capsici* 55]	ALN86700
			81	3E-64	pilus assembly protein PilZ [*Lysobacter daejeonensis* GH1-9]	KGM55504
			77	4E-57	type IV pilus assembly PilZ [*Stenotrophomonas maltophilia* R551-3]	KRG40644
			76	3E-57	type IV fimbriae assembly protein [*Xanthomonas campestris* pv. *campestris* str. ATCC 33913]	NP_636400
			73	6E-58	type IV pilus assembly protein PilZ [*Xanthomonas translucens*]	WP_003479797
