# Supplementary material for: Pea Broth Enhances the Biocontrol Efficacy of Lysobacter capsici AZ78 by Triggering Cell Motility Associated with Biogenesis of Type IV Pilus
Source: Front Microbiol. 2016 Jul 26;7:1136. doi: 10.3389/fmicb.2016.01136 (PMC4960238; doi:10.3389/fmicb.2016.01136)
Supplement: Supplementary file 5 [file Image_1.PDF]

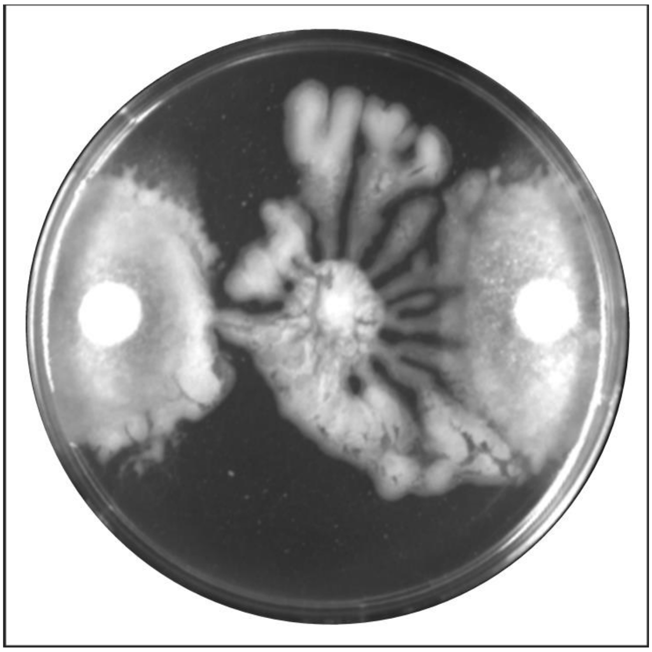

Figure S1: *Lysobacter capsici* AZ78 dendrite-like macrocolony in dual-culture assay with *Phytophthora infestans.* The dual-culture assay of *P. infestans* and *L. capsici* AZ78 (centre) on Pea Agar Medium (1.2% Agar). Images was captured using Bio-Rad Quantity One software.
