# Supplementary material for: Pea Broth Enhances the Biocontrol Efficacy of Lysobacter capsici AZ78 by Triggering Cell Motility Associated with Biogenesis of Type IV Pilus
Source: Front Microbiol. 2016 Jul 26;7:1136. doi: 10.3389/fmicb.2016.01136 (PMC4960238; doi:10.3389/fmicb.2016.01136)
Supplement: Supplementary file 6 [file Image_2.PDF]

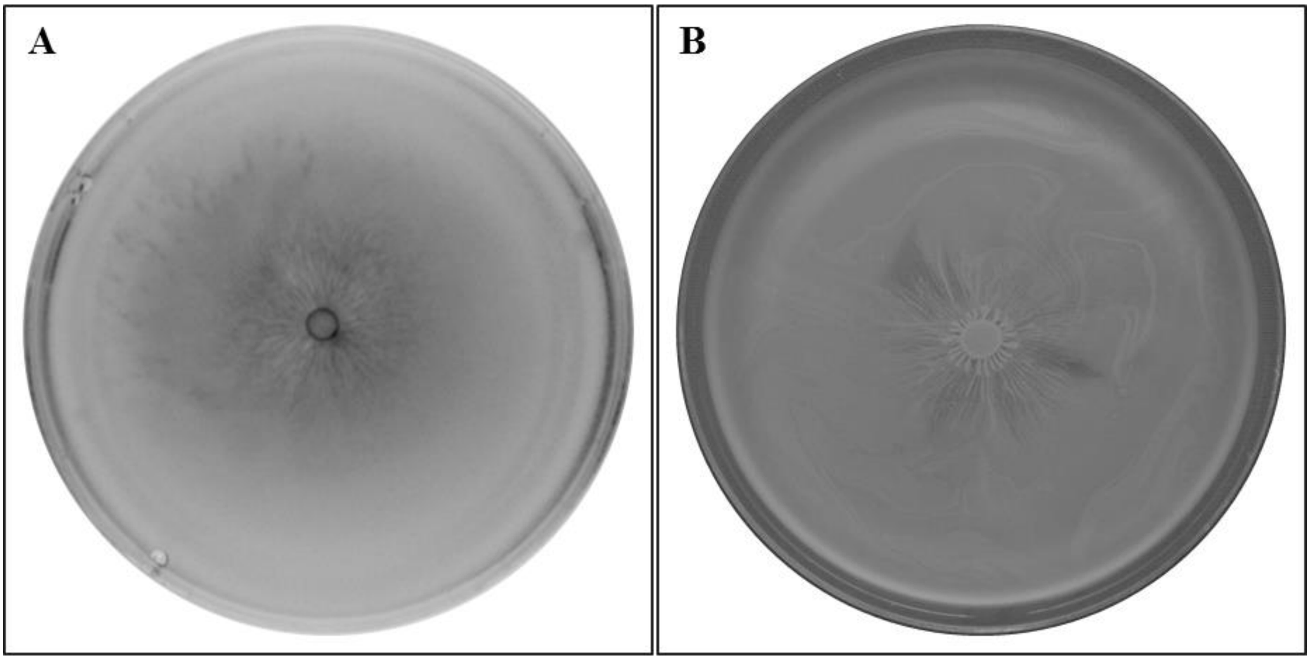

Figure S2: Morphology of *Bacillus amyloliquefaciens* S499 and *Pseudomonas chlororaphis* M71 macrocolonies in swimming motility assays. The swimming motility was evaluated on Swimming Agar (SWM). Images of macrocolonies were taken with Bio-Rad Quantity One software after 20 h incubation at 27°C. **A**, *B. amyloliquefaciens* S499; **B**, *P. chlororaphis* M71.
