# Supplementary material for: Pea Broth Enhances the Biocontrol Efficacy of Lysobacter capsici AZ78 by Triggering Cell Motility Associated with Biogenesis of Type IV Pilus
Source: Front Microbiol. 2016 Jul 26;7:1136. doi: 10.3389/fmicb.2016.01136 (PMC4960238; doi:10.3389/fmicb.2016.01136)
Supplement: Supplementary file 7 [file Image_3.PDF]

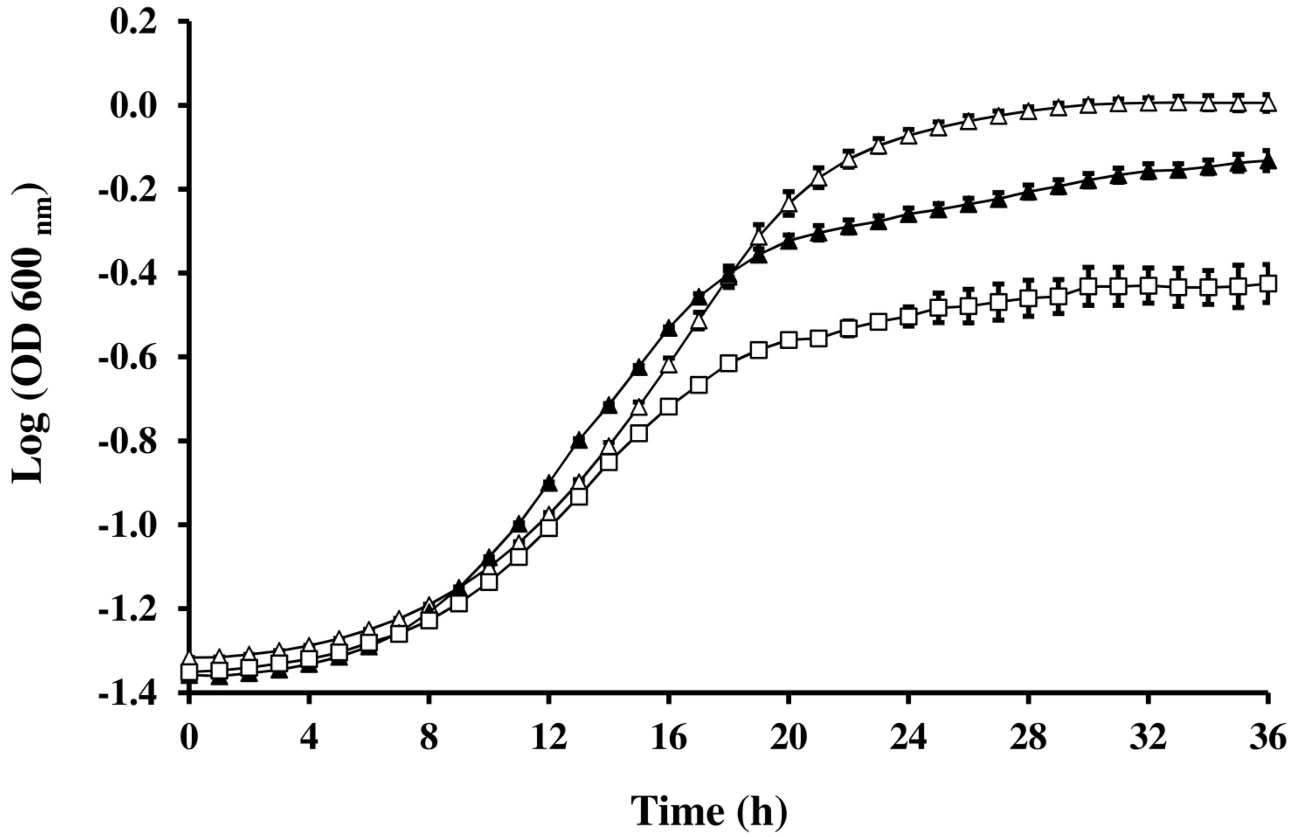

Figure S3: Growth curves of *Lysobacter capsici* AZ78 in different liquid media. Cell growth of *L. capsici* AZ78 was monitored in LB (Δ), PB (□) and SWR (▲) broth. The OD_{600} value was taken each hour for 36 h at 27°C. Not-inoculated media were used as controls. Mean and standard error values, for seven replicates pooled from two experiments, are reported for each time and media.
